# A high ATP concentration enhances the cooperative translocation of the SARS coronavirus helicase nsP13 in the unwinding of duplex RNA

**DOI:** 10.1038/s41598-020-61432-1

**Published:** 2020-03-11

**Authors:** Kyoung-Jin Jang, Seonghwan Jeong, Dong Young Kang, Nipin Sp, Young Mok Yang, Dong-Eun Kim

**Affiliations:** 1 Department of Bioscience and Biotechnology, Konkuk University, Seoul, 05029 Republic of Korea; 2 Department of Pathology, School of Medicine, Institute of Biomedical Science and Technology (IBST), Konkuk University, Seoul, 05029 Republic of Korea

**Keywords:** Biochemistry, Microbiology

## Abstract

Severe acute respiratory syndrome coronavirus nonstructural protein 13 (SCV nsP13), a superfamily 1 helicase, plays a central role in viral RNA replication through the unwinding of duplex RNA and DNA with a 5′ single-stranded tail in a 5′ to 3′ direction. Despite its putative role in viral RNA replication, nsP13 readily unwinds duplex DNA by cooperative translocation. Herein, nsP13 exhibited different characteristics in duplex RNA unwinding than that in duplex DNA. nsP13 showed very poor processivity on duplex RNA compared with that on duplex DNA. More importantly, nsP13 inefficiently unwinds duplex RNA by increasing the 5′-ss tail length. As the concentration of nsP13 increased, the amount of unwound duplex DNA increased and that of unwound duplex RNA decreased. The accumulation of duplex RNA/nsP13 complexes increased as the concentration of nsP13 increased. An increased ATP concentration in the unwinding of duplex RNA relieved the decrease in duplex RNA unwinding. Thus, nsP13 has a strong affinity for duplex RNA as a substrate for the unwinding reaction, which requires increased ATPs to processively unwind duplex RNA. Our results suggest that duplex RNA is a preferred substrate for the helicase activity of nsP13 than duplex DNA at high ATP concentrations.

## Introduction

Severe acute respiratory syndrome (SARS) is an acute respiratory infectious disease caused by a novel coronavirus (SARS-CoV or SCV) that has claimed almost 800 deaths in early 2003^1^. SCV is an enveloped, positive single-stranded RNA virus (or (+) ssRNA virus) with a genome of ~30 kb in length^2,3^. Two-thirds of the SCV genome at the 5′-end comprise replicase genes (*orf*1*ab*) encoding 16 nonstructural proteins (nsPs). The replicase genes comprising open reading frames (OFR1a and 1b) are translated into two large replicative polyproteins, pp1ab (~790 kDa) and pp1a (~490 kDa), which are involved with and without ribosomal frameshifting into the −1 frame^4,5^. These two translational polyproteins are processed autoproteolytically by the major viral cysteine proteases M^PRO^ or 3CL^PRO^ to produce 16 non-structural proteins (nsPs), including RNA-dependent RNA polymerases (RdRp, nsP12) and NTPase/helicase (nsP13)^6–8^. These viral replicases are the core of membrane-bound replication-transcription complexes that synthesize the entire viral genome and eight subgenomic mRNAs^9,10^.

Because viral helicase is considered to be essential for subsequent viral replication and proliferation, it is an important potential target for antiviral therapy^11–13^. In addition, the inhibition of these targets may interfere with the metabolism of the infecting virus without strong side effects in patients. Several viral helicases have been used as proven drug targets due to the inhibition of helicase activity in animal models of herpes simplex virus (HSV) and in the treatment of hepatitis C^14,15^. Therefore, much effort has been spent on the development of small-molecule inhibitors and chemicals as drug candidates to inhibit the function of SARS coronavirus helicase nsP13 (SCV nsP13)^16–20^. Helicases are motor proteins that unwind a double-stranded (ds) nucleic acid into two single-stranded (ss) nucleic acids using energy derived from NTP hydrolysis during translocation along a single strand for nucleic acid replication, recombination, and DNA repair^21–24^. Helicases are classified into distinct classes depending on whether they can bind ss nucleic acid, unwind dsRNA or dsDNA or both, the polarity of the unwinding (5′ to 3′ or 3′ to 5′), and whether certain signature motifs are present in the primary sequence^25–28^. The SCV helicase nsP13 can unwind both dsRNA and dsDNA with a 5′-ss tail along the polarity of 5′ to 3′^7,29^, and the enzyme can hydrolyze all deoxyribonucleotide and ribonucleotide triphosphates^6^. In a previous study, we revealed that the amplitudes of SCV helicase nsP13 are decreased as the length of duplex DNA increased in the presence of ATP^30^. Moreover, the amplitudes of duplex DNA with a short 5′-ssDNA tail were very low compared with other duplex DNAs with a long 5′-ssDNA tail. Multiple nsP13 monomers bind to more than one site along the ssDNA. SCV helicase nsP13 is derived from (+) ssRNA virus and is essential for the replication of viral RNA. Nevertheless, no study has investigated definite unwinding kinetics of duplex RNA as a substrate by nsP13. Thus, it is highly important to understand the unwinding mechanism of duplex RNA by nsP13 to develop more efficient anti-SCV drugs in depth.

In this study, we used partial duplex RNAs and DNAs as a model and control substrate, and the single-turnover kinetics of duplex RNA with a 5′-ss tail of different lengths was investigated according to various times and the concentration of ATP or nsP13. We observed that nsP13 exhibits unwinding results of different patterns in RNAs unlike DNAs. Contrary to expectation, the length of the 5′-ss tail, used as a loading strand of multiple nsP13 monomers, was not significantly related to enhancement of the RNA unwinding process. However, we demonstrated that this result is caused by the substrate specificity of nsP13. We also revealed that the unwinding process of duplex RNA by nsP13 is significantly related to the amount of ATP required for the binding, translocation, and unwinding of substrate although it is not completely clear how ATP and ATPase cycles function in the work performed by helicase nsP13. In addition, we observed that the cooperative translocation by nsP13 is also related to the amount of ATP.

## Results

### Single-turnover kinetics of duplex RNA unwinding by helicase nsP13

**Figure 1:**
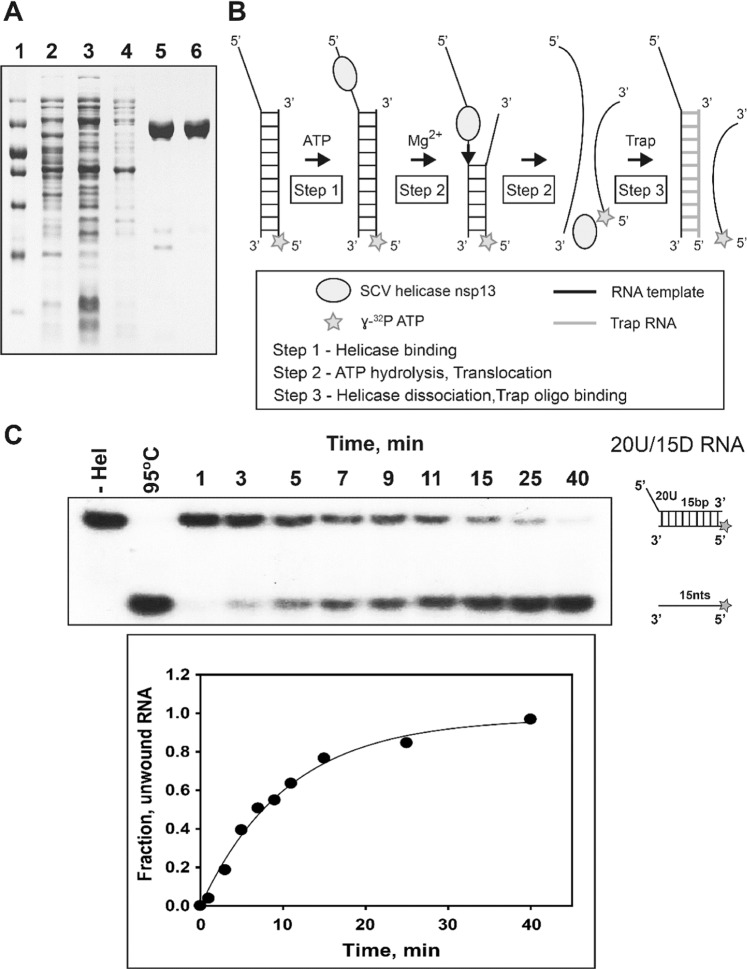
Purification and single-turnover kinetics of duplex RNA unwinding by the SCV helicase nsP13. (**A**) *Lane 1*, Protein marker, from bottom (in kilodaltons) 10, 20, 30, 40, 50 (strong density), 70, and 100. *Lane 2*, Crude cell extract without induction. *Lane 3*, Crude cell extract with IPTG induction. *Lane 4*, Proteins washed by nickel affinity chromatography. *Lane 5*, Helicase nsP13 eluted by nickel affinity chromatography. *Lane 6*, Helicase nsP13 eluted by size exclusion chromatography. (**B**) Schematic view of RNA duplex-unwinding by SCV helicase nsP13. (**C**) Representative native gel shift assay of RNA duplex unwinding by nsP13 with the 20U/15D RNA (5′-poly(U) tail of 20 bases/duplex length of 15 bp). The unwinding reaction was carried out at 37 °C for various times as described in the Materials and Methods section. The unwinding products were resolved by non-denaturing 15% PAGE. The gel was exposed to X-ray film and quantitated using image J software.

Previously, studies to identify the unwinding mechanism of duplex DNA or RNA by nsP13 were performed using multiple or single-turnover kinetics experiments^6,7,15,27^. However, although nsP13 is derived from the (+) ssRNA virus, no mechanistic study exists concerning unwinding using duplex RNA as a model substrate. In this study, we used duplex RNA as a substrate to observe the single-turnover kinetics of duplex RNA unwinding, and the results were compared with the unwinding kinetics of duplex DNA. To investigate the unwinding mechanism of duplex RNA, we purified nsP13 with high purity and activity for *in vitro* studies as described in the Materials and Methods section (Fig. 1A). A representative reaction of duplex RNA unwinding by nsP13 is shown in Fig. 1B, in which the nsP13 could be bound to the 5′-ss tail in the presence of ATP without ATP hydrolysis. Next, ATP hydrolysis, caused by adding magnesium ions, allows nsP13 to separate duplex RNA and to translocate along ssRNA unwound in a 5′ to 3′ direction (Fig. 1B). We prepared similar reaction conditions for the unwinding assay of duplex RNA as in a previous DNA study. RNA trap oligonucleotides with unlabeled bottom strands were chosen to prevent the re-initiation of unwinding by free nsP13 during unwinding (Supplementary Fig. 1,A). A large excess (0.5 µM) of trap RNA efficiently prevented re-association with duplex substrate once nsP13 falls off during the helicase reaction (Supplementary Fig. 1B). Additionally, we used two different substrates (20U/15D RNA and 20U/25D RNA) to determine the optimal concentration of nsP13. The nsP13 of 0.5 µM showed an ideal amplitude in the unwinding assay using short (15 duplex, 15D) and long (25 duplex, 25D) duplex substrates with tail-fixed 5′-poly(U) (20 nt poly(U), 20U) (Supplementary Fig. 2). We also confirmed whether ATP is required for the unwinding assay of duplex RNA, as in the case of duplex DNA (Supplementary Fig. 3). Determining the optimal conditions is critical to understand the single-turnover kinetics of nsP13 through various substrates. Therefore, 0.5 µM trap RNA, 0.5 µM nsP13, and 2 mM ATP were used in the duplex RNA standard assay, and the assay was designed to monitor the unwinding kinetics of nsP13 as described in the Materials and Methods section. The helicase nsP13 unwound the duplex RNA substrates and generated ssRNA products that were resolved by 15% non-denaturing PAGE (Fig. 1C). The kinetic time course of ssRNA accumulation was plotted and fitted to an exponential function to obtain the reaction amplitudes and unwinding rates of only the helicases that were initially bound to the RNA substrates.

### Processivity of duplex RNA unwinding by nsP13

**Figure 2:**
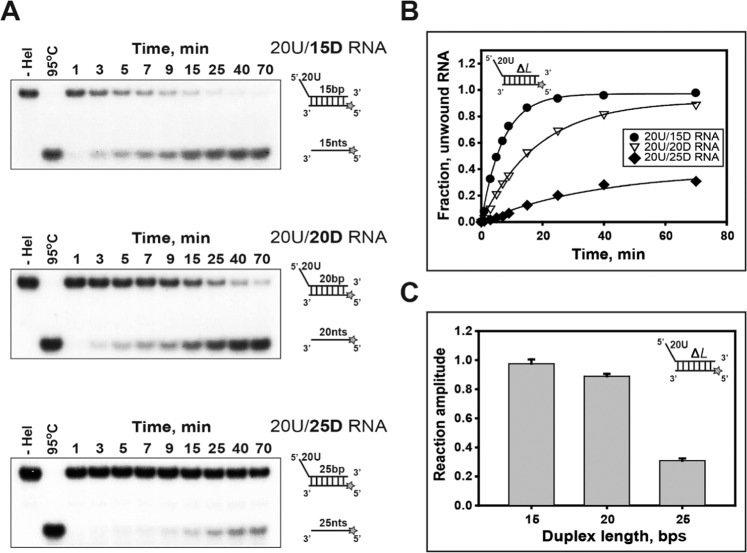
Processivity on duplex RNA substrates with different duplex lengths. (**A,B**): The single-turnover unwinding of duplex RNA substrates with different lengths of duplex. (**A**) Gel retardation assay of nsP13 and duplex RNAs with different duplex lengths. The substrates of 15 bp (15D), 20 bp (20D), and 25 bp (25D) duplexes containing a 5′-poly(U) tail of 20 bases (20U) were designed as shown in Supplementary Table 1. The unwinding products were resolved by non-denaturing 15% PAGE. (B, C) The amplitudes were as follows: 15 bp (●: 20U/15D RNA) = 0.98 ± 0.03, 20 bp (**▽**: 20U/20D RNA) = 0.89 ± 0.02, and 25 bp (◆: 20U/25D RNA) = 0.31 ± 0.02.

The processivity of RNA unwinding, single-base-pair unwinding (*P*), is defined as the probability of the separation of a base pair by helicase in contrast to helicase dissociating from that position on the RNA. It is evaluated as the distance moved by the helicase before dissociating from the RNA. Therefore, to estimate the relative processivity of nsP13 on different duplex lengths, three substrates (shown in Supplementary Table 1: 20U/15D, 20U/20D, and 20U/25D RNA) comprising progressively longer duplex regions were used, and the measured amplitudes were indicated as the evaluated processivity of nsP13. The ssRNA products unwound by nsP13 were resolved by 15% non-denaturing PAGE (Fig. 2A). In a previous study, the amplitudes were decreased with the increase in the duplex DNA length, and the maximal amplitudes of DNA containing duplexes (20D and 30D) of a short length were calculated within 3 minutes^30^. In this study, although the amplitudes in duplex RNA were also decreased with the increase in duplex length similar to the result of duplex DNA, the final amplitudes versus duplex length were very low despite the long reaction times (Fig. 2B). A different pattern in the processivity of RNA versus DNA was similar to HCV NS3^31^. Thus, these results imply that nsP13 might have a different substrate affinity for DNA or RNA, and the characteristic may be required as an important determining factor for the processivity of nsP13 in duplex unwinding.

### Inhibition of duplex RNA unwinding by increasing the length of the 5′-ss tail

A short 5′-ss tail for the loading of coronavirus helicase is critical to initiate efficient duplex substrate unwinding using the energy of ATP hydrolysis^7,29,30,32^. A previous study have shown that the quantities of the unwound ss product were increased depending on the increase in the length of the 5′-ss tail, and the minimum length of the 5′-ss tail, at least more than 5 nt long, was required for unwinding of the duplex substrate by nsP13^29,30^. We also demonstrated that the stimulation of ATPase activity is dependent on the length of ssDNA or ssRNA^33^. In addition, we observed that the accumulation of ssDNA unwound by nsP13 was increased as the length of the 5′-ssDNA tail increased, implying that multiple binding of nsP13 onto the 5′-ss tail could show enhanced processivity^30^.

**Figure 3:**
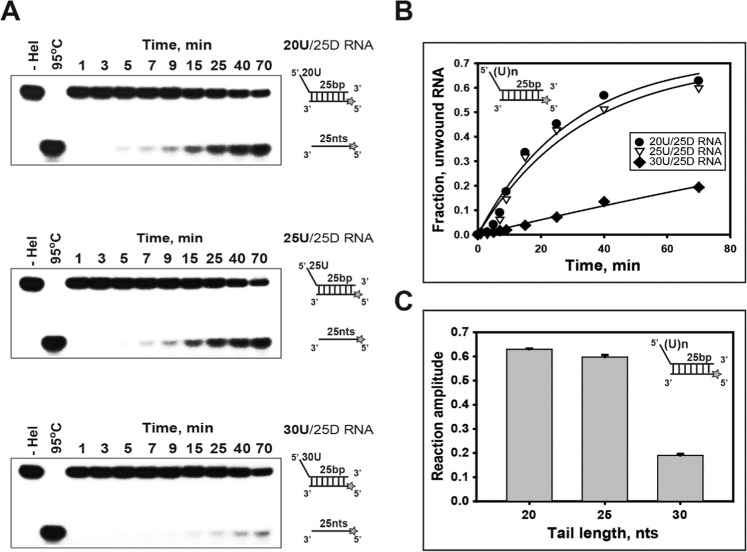
Unwinding of duplex RNA substrates with 5′-ss tails of different lengths. (**A,B**): Single-turnover unwinding of 25 bp (25D) RNA substrates with 5′-poly(U) tails of different lengths by helicase nsP13. (**A**) Gel retardation assay of nsP13 and duplex RNA substrates with 5′-ss tails of different lengths. Three different substrates containing 25 duplex-fixed (25D) and the 5′-poly(U) tail of 20 bases (20U), 25 bases (25U), and 30 bases (30U) were designed as shown in Supplementary Table 1. The unwinding products were resolved by non-denaturing 15% PAGE. (**B,C**) The amplitudes were as follows: 20U (●: 20U/25D RNA) = 0.63 ± 0.01, 25U (**▽**: 25U/25D RNA) = 0.6 ± 0.01, and 30U (◆: 30U/25D RNA) = 0.19 ± 0.01.

As shown in Supplementary Table 1, three duplex RNA substrates containing a 25 bp duplex (25D) with different lengths (20U, 25U, and 30U) of the 5′-poly(U) tail were used to observe whether duplex RNA unwinding is dependent on the length of the 5′-ss tail. Compared with duplex DNA unwinding (Supplementary Fig. 4), the quantity of ssRNA unwound from dsRNA was decreased as the length of the 5′-ss tail increased (Fig. 3A,B). The amplitudes were decreased as the length of the 5′-ss tail increased on duplex RNA unwinding (Fig. 3C). In a previous study, we proposed that more ssDNA was accumulated from duplex DNA substrates that can bind more than one nsP13 molecule on the 5′-ss tail, suggesting that multiple nsP13 molecules might exhibit higher processivity along ssDNA^30^. We also suggested that the tighter binding affinity of nsP13 might be explained as an increase in unwinding with an increasing 5′-ss tail. In addition, we performed RNA unwinding assay with nsP13 of low concentration (0.2 μM) unlike dsRNA standard assay. Although weak unwinding was observed due to low concentration of nsP13, the accumulation of unwound ssRNA was slightly higher with an increasing 5′-ss tail (Supplementary Fig. 5). These results suggest that the proper loading of nsP13 for the length of 5′-ss tail may be required for cooperative translocation in RNA unwinding, but over-loading of nsP13 onto the 5′-ss tail increased may rather inhibit translocation of nsP13 in RNA unwinding compared with DNA, which might imply the helicase according to substrate have different substrate specificity such as binding affinity.

### Substrate specificity of nsP13

Most helicases specifically separate only one type of substrate, either duplex DNA or RNA^34,35^. However, some helicases, such as HCV NS3, SVG large T antigen, and human UPF1, could unwind both duplex DNA and RNA^36–38^, mostly displaying strong apparent discrimination for the nucleic acid of a particular class. The basis for this discrimination is unknown, and it is very important to understand how viral replication is controlled.

**Figure 4:**
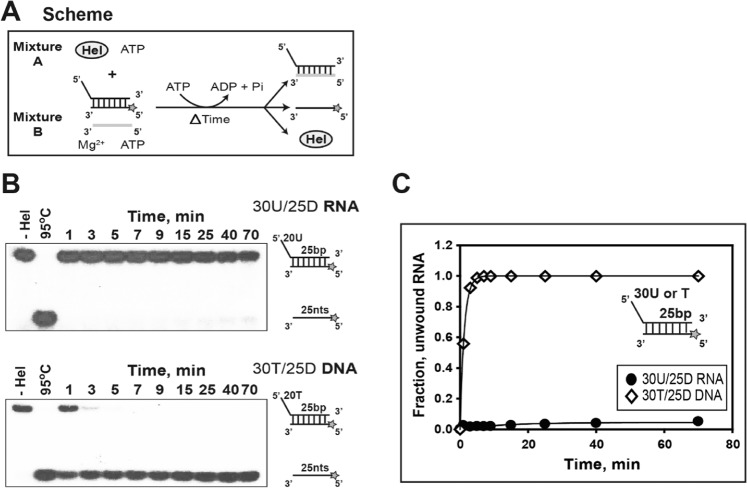
Substrate specificity of helicase nsP13 in the unwinding of duplex substrates. (**A**) Experimental method. (**B**) Gel retardation assay. Two different substrates of duplex RNA (30U/25D RNA) and duplex DNA (30 T/25D DNA) were designed as shown in Supplementary Table 1. Unlike the standard RNA unwinding method, helicase nsP13 (0.5 μM) and substrate (5 nM) were separately prepared as shown in Fig. 4A. each equal volume of mixture A [helicase nsp13 (0.5 μM), 50 mM Tris-HCl (pH 6.8), 50 mM NaCl, 2 mM ATP, and 10% glycerol] and mixture B [ɣ-^32^P-labeled duplex substrates (5 nM), 5 µM trap oligo (unlabeled bottom strand), 2 mM ATP, and 13 mM MgCl_2_] were separately made. Mixture A and B were immediately mixed without preincubation and reacted at 37 °C. The unwinding products were resolved by non-denaturing 15% PAGE. (**C**) The amplitudes were as follows: 30U/25D RNA (●) = 0.04 ± 0.003 and 30 T/25D DNA (◇) = 1.02 ± 0.02.

To understand substrate specificity of nsP13 in unwinding process, we separately prepared nsP13 and duplex substrate (30U/25D RNA or 30 T/25D DNA) to block the preloading of nsP13 onto the 5′-ss loading strand unlike the standard single-turnover condition used in this study. Also, trap oligo was separately prepared from nsP13. The unwinding reaction was immediately initiated by mixing mixture A (nsP13) and mixture B (duplex substrate and trap oligo) without pre-incubation at 37 °C (Fig. 4A). This experiment was conducted to compare the relative translocation of nsP13 against cognate RNA and DNA substrates containing the same sequence (except for the presence of U versus T) without preloading. The substrates were controlled for the effects of the tail length, tail sequence, and duplex length. The Fig. 3 showed that 30U/25D RNA was separated into ssRNA to more than 30% completion. However, in the experiment that blocked the preloading of nsP13 onto the 5′-ss loading strand, 30U/25D RNA was unwound almost to incompletion (~5%; Fig. 4B,C), implying that preloading of nsP13 is strongly needed to unwind duplex RNA. Unlike the case of duplex RNA, 30 T/25D DNA was unwound almost to completion regardless of preloading onto the 5′-ss tail by nsP13 (Fig. 4B,C). These results suggest that the early stages of reaction initiation may be more favorable on the DNA substrate. Also, these results propose that nsP13 could distinguish either the RNA or DNA substrate, and there is different unwinding activity depending on the substrate. The difference of unwinding might be caused by different binding affinity depending on the substrate, which may exhibit the difference of translocation in unwinding process. Therefore, if nsP13 has a higher affinity to RNA than to DNA, it might show to translocate on ssDNA much faster than ssRNA in unwinding process.

**Figure 5:**
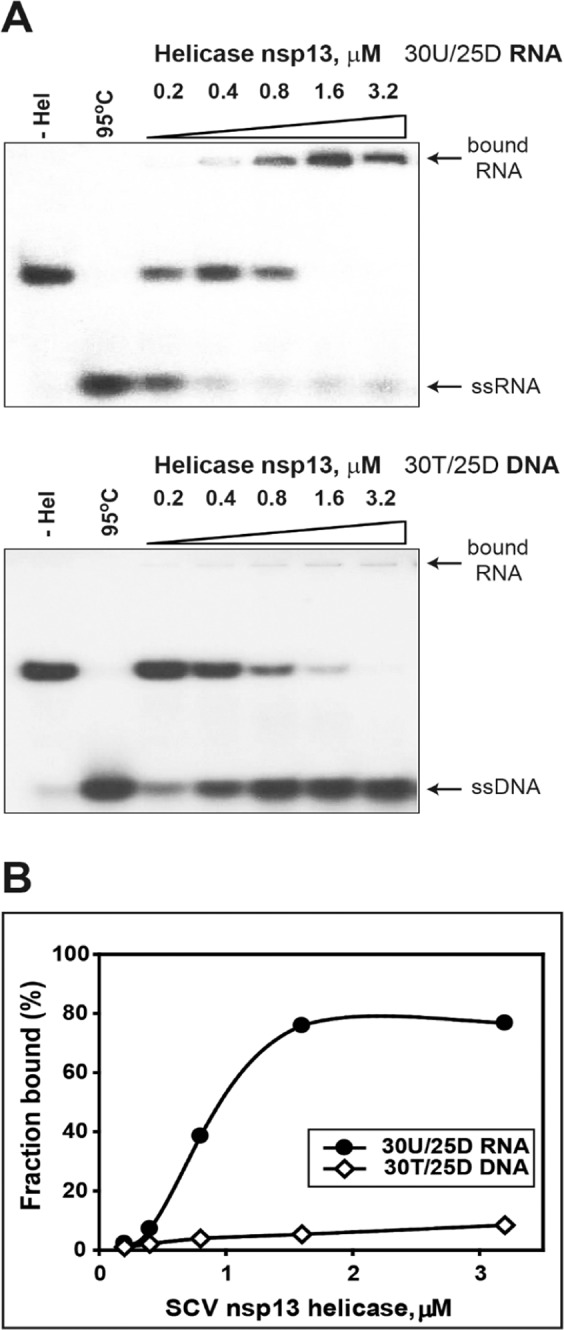
Difference in the substrate-dependent binding affinity of helicase nsP13. (**A**) Gel shift assays using helicase nsP13 of various concentrations; 5 nM of each substrate (30U/25D RNA and 30 T/25D DNA) was prepared with various concentrations of helicase nsP13 (0.2, 0.4, 0.8, 1.6, and 3.2 μM) in mixture A. The unwinding reaction was immediately initiated by mixing mixture A and mixture B (with trap oligos) as described in the EMSA method. For a single-time assay, the reactions were carried out at 37 °C for 1 hr. The unwinding products were resolved by non-denaturing 15% PAGE. (**B**) Combined percentage of helicase nsP13 against RNA and DNA duplex substrates.

To further understand the difference of unwinding activity depending on substrate, the gel shift assay was conducted with two different substrates (30U/25D RNA and 30 T/25D DNA) and nsP13 in a dose-dependent manner (Fig. 5A). The bands with significant retardation corresponding to a high molecular mass of nsP13 molecules were seen only in the duplex RNA substrate (30U/25D RNA), and the intensity of which increased with the rising protein concentration. However, in the case of duplex DNA, the DNA-protein complexes of low intensity were seen in the higher molecular mass region through increasing the nsP13 concentration. Besides, duplex RNA unwinding under this condition was extremely poor compared with unwinding of a similar DNA substrate. The intensities of the bands corresponding to the major substrate-protein complex at various protein concentrations in the case of duplex RNA and DNA were quantified by densitometric scanning, and the corresponding substrate binding activities (%) were plotted accordingly. From this analysis, the RNA-protein complexes exhibited up to 80% substrate binding activity compared with DNA-protein complexes (up to 8%) (Fig. 5B). Binding of ATP may cause the conformational changes of helicase, which affects their substrate binding^39,40^. Additionally, we conducted the gel shift assay using three dsRNA substrates (20U, 25U, and 30U/25D RNA) in a nsP13 dose-dependent manner without trap oligos. In this experiment, the bands with significant retardation were increased depending on the increase in the length of the 5′-ss tail (Supplementary Fig. 6). This result suggests that the strong binding affinity of nsP13 onto RNA could be explained as a decrease in unwinding and translocation with an increasing 5′-ss tail.

### Increased ATP requirement for duplex RNA unwinding by nsP13

The unwinding of duplex substrates by helicase is a stepwise process that comprises helicase binding, translocation, and local separation of duplex substrates. The nsP13 prefers ATP as an energy source^7,30^, and the essential activity of nsP13 is one-way translocation driven by the ATPase cycles^33^. Each cycle of the ATPase reaction requires basic steps containing ATP binding, hydrolysis, phosphate release, ADP release, and rebinding of ATP. Therefore, the amount of ATP might influence the unwinding mechanism by nsP13. In a previous experiment using duplex DNA, although there were differences depending on the length of the 5′-ss tail and duplex DNA, nsP13 showed high unwinding activity in the same concentration of ATP. However, in this study, duplex RNA unwinding by nsP13 exhibited very poor unwinding activity compared with duplex DNA of the same size and sequence (except for the presence of U versus T) in the same concentration of ATP. These results might strongly imply that nsP13 has different ATP demands depending on the type of substrate in the unwinding process.

**Figure 6:**
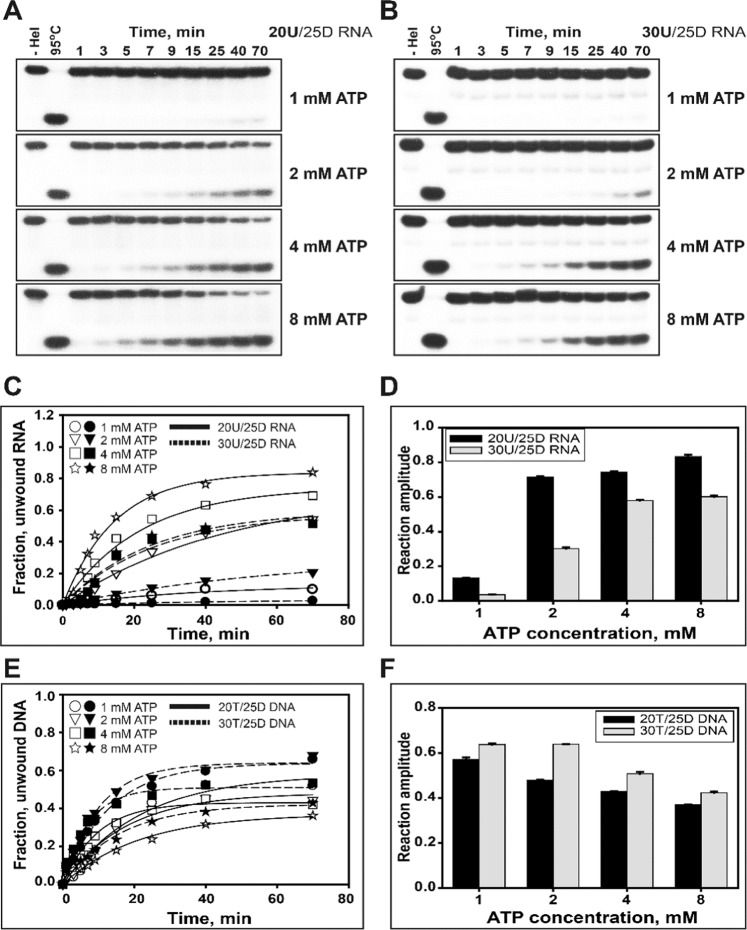
Additional ATP requirement to promote ATP-dependent translocation of helicase nsP13. (**A,B**): Gel retardation assay of helicase nsP13 and duplex RNA substrates. The unwinding of 20U/25D RNA and 30U/25D RNA or 20 T/25D DNA and 30 T/25D DNA was reacted with various concentrations of ATP (1, 2, 4, and 8 mM). The unwinding reaction was performed with 0.5 μM nsP13 (for RNA) or 0.1 μM nsP13 (for DNA) and each 5 nM substrate at 37 °C as described in the standard unwinding method. The unwinding products were resolved by non-denaturing 15% PAGE. (**C,D**) The amplitudes for RNAs were as follows: ①20U/25D RNA (Solid line) – 1 mM ATP (○) = 0.13 ± 0.001, 2 mM ATP (**▽**) = 0.71 ± 0.005, 4 mM ATP (□) = 0.74 ± 0.005, and 8 mM ATP (☆) = 0.83 ± 0.01; ②30U/25D RNA (Dotted line) – 1 mM ATP (●) = 0.03 ± 0.001, 2 mM ATP (▼) = 0.3 ± 0.01, 4 mM ATP(■) = 0.58 ± 0.003, and 8 mM ATP (★) = 0.6 ± 0.01. (E, F) The amplitudes for DNAs were as follows: ①20 T/25D DNA (Solid line) – 1 mM ATP (○) = 0.57 ± 0.01, 2 mM ATP (**▽**) = 0.48 ± 0.001, 4 mM ATP (□) = 0.43 ± 0.001, and 8 mM ATP (☆) = 0.37 ± 0.002; ②30 T/25D DNA (Dotted line) – 1 mM ATP (●) = 0.64 ± 0.004, 2 mM ATP (▼) = 0.64 ± 0.001, 4 mM ATP (■) = 0.51 ± 0.01, and 8 mM ATP (★) = 0.42 ± 0.01.

To distinguish the ATP amount required according to the type of substrate, two different duplex RNA substrates (20U/25D and 30U/25D RNA) were used in this experiment. For the helicase reaction, 5 nM of duplex substrates and 0.5 µM of nsP13 were used with various ATP concentrations (1, 2, 4, and 8 mM) to assess the unwinding effect by increasing the ATP concentration (Fig. 6A–D). Although the RNA unwinding process was not more effective than DNAs at 2 mM ATP, the ssRNA accumulation and amplitude increased as the ATP concentration increased from 1 mM to 8 mM ATP (Supplementary Fig. 4 and Fig. 6C,D). Cooperative translocation by nsP13 was not exhibited by the difference in the length of the 5′-ss tail with ATP in a dose-dependent manner, but the quantity of ssRNA unwound from 30U/25D RNA was increased up to ~55% with 8 mM ATP. These results imply that more ATPs are required for the efficient unwinding and translocation of nsP13 in duplex RNA unwinding. In Fig. 5 and Supplementary Fig. 6, the RNAs were clearly bound more tightly than DNA. From these results, the issue most likely relates to the rate of ATP-dependent translocation, which is faster on DNA at low ATP. There is a distinction between binding and translocation. Rapid translocation will allow the enzyme to rapidly dissociate from the RNA.

To further examine the unwinding effect with an increased ATP concentration, the concentration of nsP13 was determined for the duplex DNA substrates (Supplementary Fig. 7), and we prepared duplex DNA substrates (20 T/25D and 30 T/25D DNA) for comparative studies of duplex RNA unwinding. To compare the time course of duplex RNA unwinding, 0.1 µM nsP13 and different concentrations of ATP were used for duplex DNA unwinding. Duplex DNA substrates were separated by a 15% non-denaturing PAGE (Supplementary Fig. 8). The quantities of the ssDNA product unwound from two DNA substrates were decreased depending on the increased ATP concentrations unlike those from duplex RNA substrates (Fig. 6E). In addition, a decrease in the amplitude of DNA unwinding was observed with an increased ATP concentration (Fig. 6F). These results suggest that nsP13 with relatively low binding affinity to DNA substrate might mostly use the chemical energy of ATP hydrolysis for the translocation and local separation of duplex substrates. Thus, we propose that high ATP concentrations enhance the accumulation of ssRNA unwound by nsP13, but interfere in the case of duplex DNA, implying that a faster translocation of nsP13 by more ATP concentrations might be easily dissociated from DNA substrates.

### Enhanced cooperative translocation in duplex RNA unwinding by increasing nsP13 with high-dose ATP

Helicases are proposed to be active as monomers, dimers, or oligomers to translocate along ss nucleotides and unwind duplex substrates^41–43^. In a previous study, we observed the oligomerization of nsP13 by chemical cross-linking using DMS^30^. A previous study showed that both the ATPase and helicase activities of HCV NS3 were enhanced in a protein dose dependence. This result is explained by the unwinding mechanism containing transient dimeric molecules by NS3^44^. In addition, optimal unwinding by NS3 was dependent on the protein concentration to form multiple monomers of NS3^45^.

**Figure 7:**
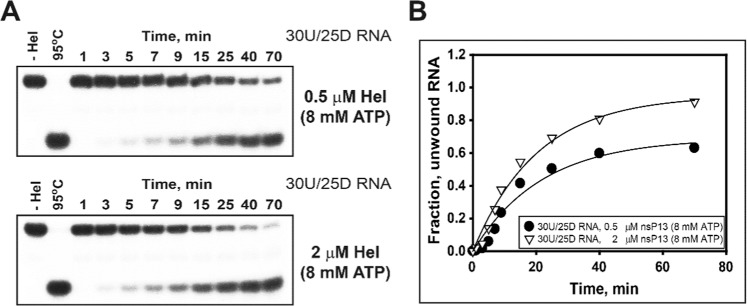
Enhanced duplex RNA unwinding by the cooperative translocation of helicase nsP13 under adequate ATP concentrations. (**A**) Gel retardation assay of nsP13 and duplex RNA substrates. The unwinding of 30U/25D RNA with 0.5 μM and 2 μM helicase nsP13. The unwinding reaction was performed with 8 mM ATP and 5 nM substrate at 37 °C. The unwinding products were resolved by non-denaturing 15% PAGE. (**B**) The amplitudes were as follows: 0.5 μM helicase nsP13 (●) = 0.68 ± 0.01 and 2 μM helicase nsP13 (**▽**) = 0.96 ± 0.005.

To better understand the cooperative helicase activity in different protein concentrations, one duplex RNA substrate (5 nM, 30U/25D RNA) was used in this experiment. The substrate was incubated at two different nsP13 concentrations (0.5 µM and 2 µM), and the reaction was initiated by the addition of 8 mM ATP, 13 mM MgCl_2_, and 5 µM trap oligo. The time courses of the unwinding reactions are shown in Fig. 7A. The unwinding kinetics were fit to a single exponential, which provided the unwinding rates. The quantity of unwound duplex RNA was increased at the high concentration of nsP13 (2 µM nsP13) using 8 mM ATP (Fig. 7A). Furthermore, a high concentration of nsP13 allowed the enzyme to maximally unwind duplex RNA up 95% (Fig. 7B). These results suggest that high-dose ATPs may be required to enhance cooperative translocation by multiple nsP13 monomers on RNA substrates, explaining that these unique activities in RNA unwinding result from the SCV helicase nsP13 itself.

## Discussion

SCV helicase nsP13 unwinds both DNA and RNA substrates using the energy of ATP hydrolysis with 5′ to 3′ polarity^6,7,46^. We observed that nsP13 could not unwind duplex RNA efficiently compared with that in a previous study using a DNA substrate under the same single-turnover condition although it is a known RNA helicase^30^. Additionally, we also identified that duplex DNA was unwound almost to completion, but the unwinding of duplex RNA was very poor under the same conditions. These results indicate that RNA helicase nsP13 is more favorable on duplex DNA for duplex unwinding than its duplex RNA counterpart. These findings are similar to the mechanism previously suggested for NPH-II RNA helicase and HCV NS3 RNA helicase^31,47^. Despite using a higher concentration of nsP13 and a longer reaction time in this study, nsP13 exhibited poorer processivity in RNA substrates than the previous result using DNA substrates. More importantly, given that nsP13 is essential for the viral RNA replication of the SARS coronavirus, these results suggest that nsP13 requires many non-structural proteins such as RNA-dependent RNA polymerase (RdRp) to enhance its processivity activity toward RNA substrates. Recently, the analysis of single-turnover kinetics exhibited that SCV nsP13 alone has a low processivity for RNA unwinding but a 2-fold higher helicase activity in the presence of RdRp^29^. Although nsP13 may have different affinities depending on RNA and DNA may be a determining factor for the processivity of nsP13 in duplex unwinding, more intensive experiments to monitor its affinities toward DNA or RNA are needed to understand the difference in the processivity.

In a previous study, we demonstrated the unwinding of duplex DNA by increasing the length of the 5′-ss tail. These data suggested that multiple nsP13 monomers binding on the 5′-ss tail are necessary for efficient DNA unwinding. Therefore, the increase in helicase activity with increasing 5′-ss tail may be explained by a model of functional protein interaction of unwinding described in terms of an inchworm model^48^. However, in this study using duplex RNAs, the unwinding of nsP13 was decreased as the length of the 5′ ss tail increased. nsP13 showed almost no unwinding efficiency on RNA with a 5′-ss tail of 30 nts compared with that on DNA. More surprisingly, we demonstrated that nsP13 is more favorable on DNA in the early stages of reaction initiation and binds DNA substrates faster than RNA substrates of the same sequence. HCV NS3 RNA helicase also showed poor processivity on RNA than DNA^31^, and some observations revealed that the loading strand of the hybrid substrate was chosen for the unwinding efficiency of the helicase in the single-turnover condition^47,49^. For these reasons, although RNA helicase nsP13 can unwind both duplex RNA and DNA, their different affinities for RNA and DNA may also be a key factor to determine nucleic acid unwinding and translocation by nsP13 in unwinding of the duplex. We also demonstrated that although nsP13 interacts with both DNA and RNA, nsP13 shows different substrate specificity for either DNA or RNA. The nsP13 and RNA complexes increased as the concentration of nsP13 increased, and the accumulation of free unwound ssRNA was decreased. By contrast, while free unwound ssDNA increased as the concentration of nsP13 increased, the helicase and DNA complexes were decreased. These results suggest that RNA helicase nsP13 has higher binding affinity to RNA than to DNA. Previous studies demonstrate that binding of ATP to helicase may cause the conformational changes for their substrate binding^39,40^. To further understand RNA binding affinity of nsP13, we performed the gel shift assay with three duplex RNA substrates (20U/25D, 25U/25D, and 30U/25D RNA) without trap oligos. In a nsP13 dose-dependent manner without trap oligos, the bands with significant retardation were increased depending on the increase in the length of the 5′-ss tail. This substrate specificity of nsP13 was exhibited as very low ability to unwind and translocate RNA substrates compared to DNA substrates. This similar result was also previously observed for HCV NS3^31,50,51^.

**Figure 8:**
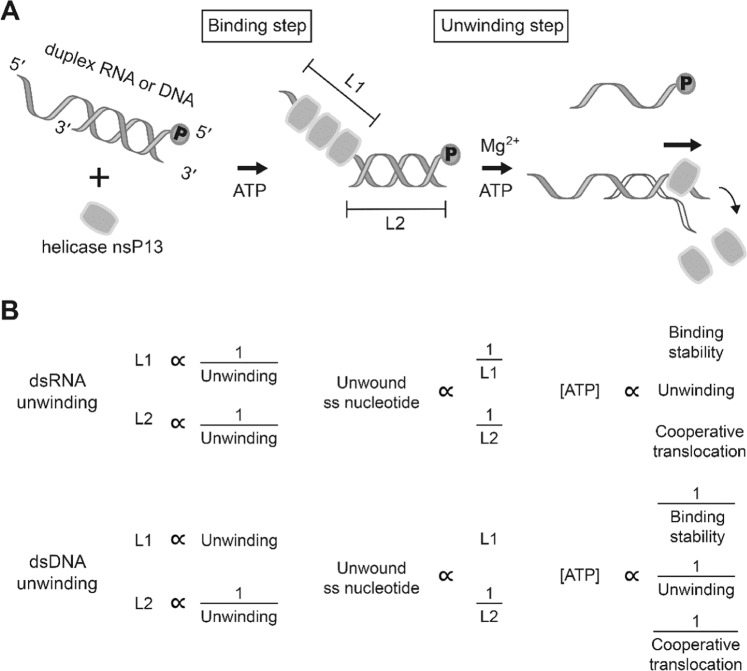
Proposed models of duplex RNA and DNA unwinding by nsP13 using various parameters. (**A**) Representative unwinding process by SCV helicase nsP13. (**B**) Unwinding characteristics of helicase nsP13 using various parameters (L1: length of 5′-ss tail; L2: length of duplex).

From our nsP13 studies on RNA and DNA unwinding, we thought that the underlying cause was the difference in the binding affinity between RNA and DNA. Because DNA is less thermodynamically stable than RNA of the same sequence and is different in physical structure and electrostatic properties^52–55^, it might be important to identify whether the difference in the thermodynamic stability determines the unwinding and processivity. However, a previous study demonstrated that the unique processivity of HCV NS3 helicase on DNA is not caused by the thermodynamic differences between RNA and DNA duplexes^31^. Most non-ring-shaped (or monomeric) SF1 helicase, such as SCV helicase nsP13, comprises two nucleic acid binding sites, RecA-like domains, in the motor core domains. Helicase exists in open and closed conformations and cycling between the two is necessary for translocation. Eventually, a conformational change in the helicase is caused upon ATP binding. Upon ATP hydrolysis and/or ADP and phosphate release, the helicase relaxes to the initial conformation, resulting in coordinated domain opening and translocation of the helicase^56^. Therefore, the closed state of helicase is populated upon ATP binding, and the open state is populated when the ATPase products dissociate. These reactions, a cycle of ATP binding and hydrolysis, lead to a stepping movement of the helicase along the direction of unwinding. Previous studies demonstrated that HCV NS3 show a weaker affinity for binding to substrates in the closed state than the open state^57,58^. As shown above, we observed that nsP13 exhibits higher binding affinity and lower unwinding activity for RNA substrates than DNA substrates in the same ATP concentration. These characteristics may be explained by different ATP demand of nsP13 depending on the substrate. Also, these results may imply that the open state of nsP13 binds with a higher affinity to RNA than to DNA and hence requires higher concentrations of ATP to drive it into the closed state. We demonstrated that the length of the 5′-ss tail does not enhance duplex RNA unwinding, but the accumulation of unwound ssRNA increases as the ATP increase in a concentration-dependent manner. Moreover, at high ATP concentrations, nsP13 was more active at a high concentration of helicase, implying enhanced cooperative translocation. This result suggests that the unwinding of duplex RNA by nsP13 is a considerably energy-consuming reaction, which also implies the requirement of higher ATP concentration for its open and closed states or stable binding to ssRNA in translocation process. Although additional experiments using ADP is required for measuring DNA and RNA affinity in binding and translocation process, cooperative translocation of nsP13 in ssRNA may be expected to require higher ATPs for stable open and closed state of nsP13 than that of ssDNA. By contrast, although enhancement of duplex DNA unwinding was observed by the increase in the 5′-ss tail, a high ATP concentration was attributable to the decrease in helicase activity, implying that the excessive activity by a high concentration of ATP causes binding instability onto the substrate. Also, these results imply that an unnecessary high ATP concentration in ssDNA leads to excess open and closed structural conformation and cycling between the two, which imply easy slipping of nsP13 from the substrate (Fig. 8). The difference of ATP demand depending on substrate might influence the unwinding, translocation, and dissociation of nsP13 from the substrate. Thus, we propose that SARS coronavirus nsP13 may require more ATPs to promote stable helicase translocation necessary for delicate RNA replication.

## Materials and Methods

### Protein expression and purification

The gene encoding the SCV helicase nsp13 domain was kindly provided by Dr. Huang, J.-D. University of Hong Kong, China. The SCV helicase nsp13 was expressed in *E. coli* Rosetta^TM^ competent cells (Novagen, Madison, WI, USA) and was purified as described previously^16^.

### Duplex substrates

RNA and DNA oligonucleotides were commercially purchased from Integrated DNA Technologies (Coralville, IA, USA) and were purified by denaturing polyacrylamide gel electrophoresis (PAGE). The single-stranded oligo fragments were radiolabeled at the 5′-end with T4 polynucleotide kinase (10 U; Takara, Tokyo, Japan) and [ɣ-^32^P]-ATP (6,000 Ci/mmol; GE Healthcare, Chicago, IL, USA). The 5′-end labeled single-stranded oligonucleotides were purified using Micro Bio-Spin^TM^ columns (BIO-RAD, Hercules, CA, USA). The partial duplex substrates (RNA and DNA) used in this study are shown in Supplementary Table 1 and were prepared as described previously^30^.

### Helicase-mediated unwinding assays

For the standard RNA unwinding assay, each equal volume of mixture A [helicase nsp13 (0.5 μM), ɣ-^32^P-labeled duplex substrates (5 nM), 50 mM Tris-HCl (pH 6.8), 50 mM NaCl, 2 mM ATP, and 10% glycerol] and mixture B [2 mM ATP, 13 mM MgCl_2_, and 5 µM trap oligo (unlabeled bottom strand)] were preincubated for 15 min at 37 °C, and then the unwinding reaction was initiated by mixing the two reaction mixtures. Additionally, different concentrations of helicase nsp13 were used in duplex DNA unwinding (0.2 μM) and Supplementary Fig. 7 (0.1 μM), and the other experimental conditions were same as the standard RNA unwinding assay. For the substrate specificity assay of nsP13, each equal volume of mixture A [ɣ-^32^P-labeled duplex substrates (5 nM), 5 µM trap oligo (unlabeled bottom strand), 50 mM Tris-HCl (pH 6.8), 50 mM NaCl, 2 mM ATP, and 10% glycerol] and mixture B [helicase nsp13 (0.5 μM), 2 mM ATP, and 13 mM MgCl_2_] were separately made. Mixture A and B were immediately mixed without preincubation and reacted at 37 °C. All the reactions were performed at 37 °C for various times, and the reactions were quenched by the addition of an equal volume of quenching solution (100 mM EDTA, 0.4% sodium dodecyl sulfate, 20% glycerol, and 0.1% bromophenol blue). The released single-stranded oligonucleotide (ssRNA or ssDNA) and unwound double-stranded duplex (dsRNA or dsDNA) were resolved by 15% non-denaturing PAGE. The control for measuring maximum unwinding was produced by heating duplex substrates for 5 min at 95 °C and loading immediately on the gel. The gel was exposed to X-ray film, and the band intensities representing the unwound strand were quantified using Image J software. The fraction of unwound RNAs or DNAs was calculated as described previously^30^, and the single-exponential equation [Eq. 1] was used to fit the unwinding kinetics: *F(t)* = A (A – exp(–k_1*_t)) (1), where F(*t*) is the fraction of unwound at time *t*, A is the amplitude of unwinding, and *k*_*1*_ is the observed rate constant of the burst phase.

### Electrophoretic mobility shift assay (EMSA)

To conduct gel shift assay, various concentrations of helicase nsP13 were added to mixture A [ɣ-^32^P-labeled duplex substrates (5 nM), 50 mM Tris-HCl (pH 6.8), 50 mM NaCl, 2 mM ATP, and 10% glycerol], and each equal volume of mixture A and B mixture B [2 mM ATP, 13 mM MgCl_2_, and 5 µM trap oligo (unlabeled bottom strand), or without trap oligo] were preincubated for 15 min at 37 °C. After preincubation, the mixture A unwinding reaction was initiated by mixing mixture B at 37 °C for 1 hr. The reaction was stopped by an equal volume of quenching solution (100 mM EDTA, 20% glycerol, and 0.1% bromophenol blue). The protein-substrate complexes were then analyzed using the gel shift assay by 15% (with trap oligos) or 10% (without trap oligos) non-denaturing PAGE. The protein-substrate complexes were then analyzed using the gel shift assay by 10% non-denaturing PAGE. The gel was exposed to X-ray film, and the band intensities representing the bound substrate were quantified using Image J software.

## Supplementary information

Supplement information.
