# Supplementary material for: A high ATP concentration enhances the cooperative translocation of the SARS coronavirus helicase nsP13 in the unwinding of duplex RNA
Source: Sci Rep. 2020 Mar 11;10:4481. doi: 10.1038/s41598-020-61432-1 (PMC7066239; doi:10.1038/s41598-020-61432-1)

**Supplementary Files**

**A high ATP concentration enhances the cooperative translocation of the SARS coronavirus helicase nsP13 in the unwinding of duplex RNA**

**Kyoung-Jin Jang^1,2^, Seonghwan Jeong^1^, Dong Young Kang^2^, Nipin Sp^2^, Young Mok Yang^2,^* and Dong-Eun Kim^2,^***

^1^Department of Bioscience and Biotechnology, Konkuk University, Seoul 05029, Republic of Korea

^2^Department of Pathology, School of Medicine, Institute of Biomedical Science and Technology (IBST), Konkuk University, Seoul 05092, Republic of Korea

*To whom correspondence should be addressed. Dong-Eun Kim, Tel: +82-2-2049-6062; Fax: +82-2-3436-6062; E-mail: kimde@konkuk.ac.kr or Young Mok Yang, Tel: +82-2-2030-7839; Fax: +82-2-2049-6192; E-mail: ymyang@kku.ac.kr

**Figure 1.** Purification and single-turnover kinetics of duplex RNA unwinding by the SCV helicase nsP13.

1. nsP13 purification


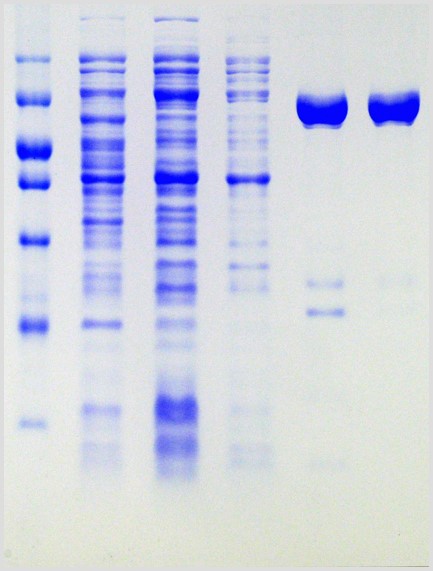


1. Gel retardation assay of nsP13 and duplex RNA substrates

**
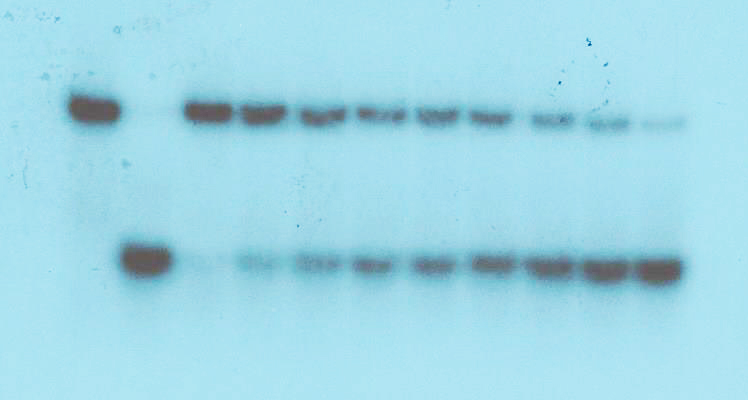
**

**Figure 2.** Processivity on duplex RNA substrates with different duplex lengths.

Gel retardation assay of nsP13 and duplex RNA substrates (20U/15, 20, and 25D RNA)


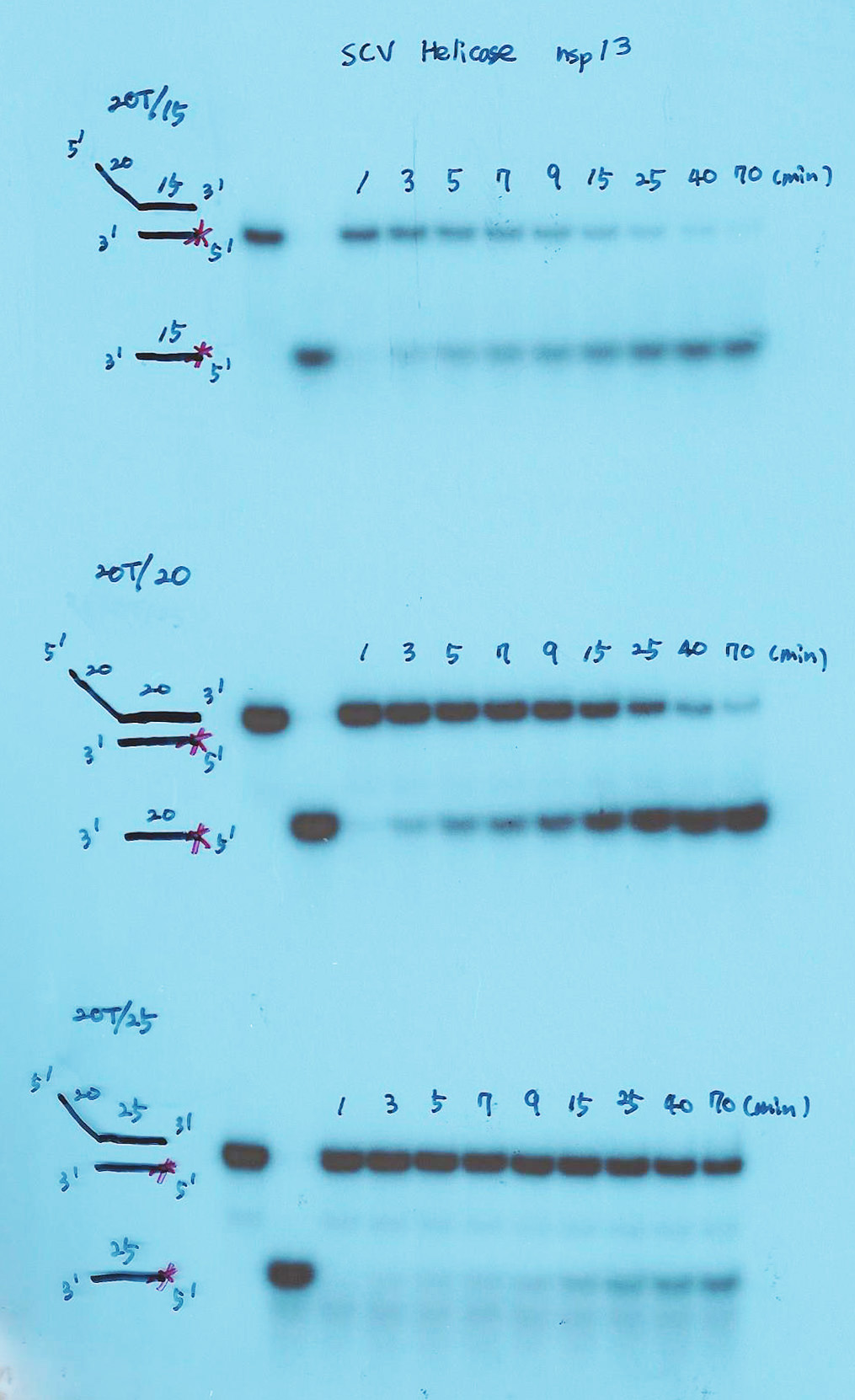


**Figure 3.** Unwinding of duplex RNA substrates with 5′-ss tails of different lengths.

Gel retardation assay of nsP13 and duplex RNA substrates (20U, 25U, and 30U/25D RNA)


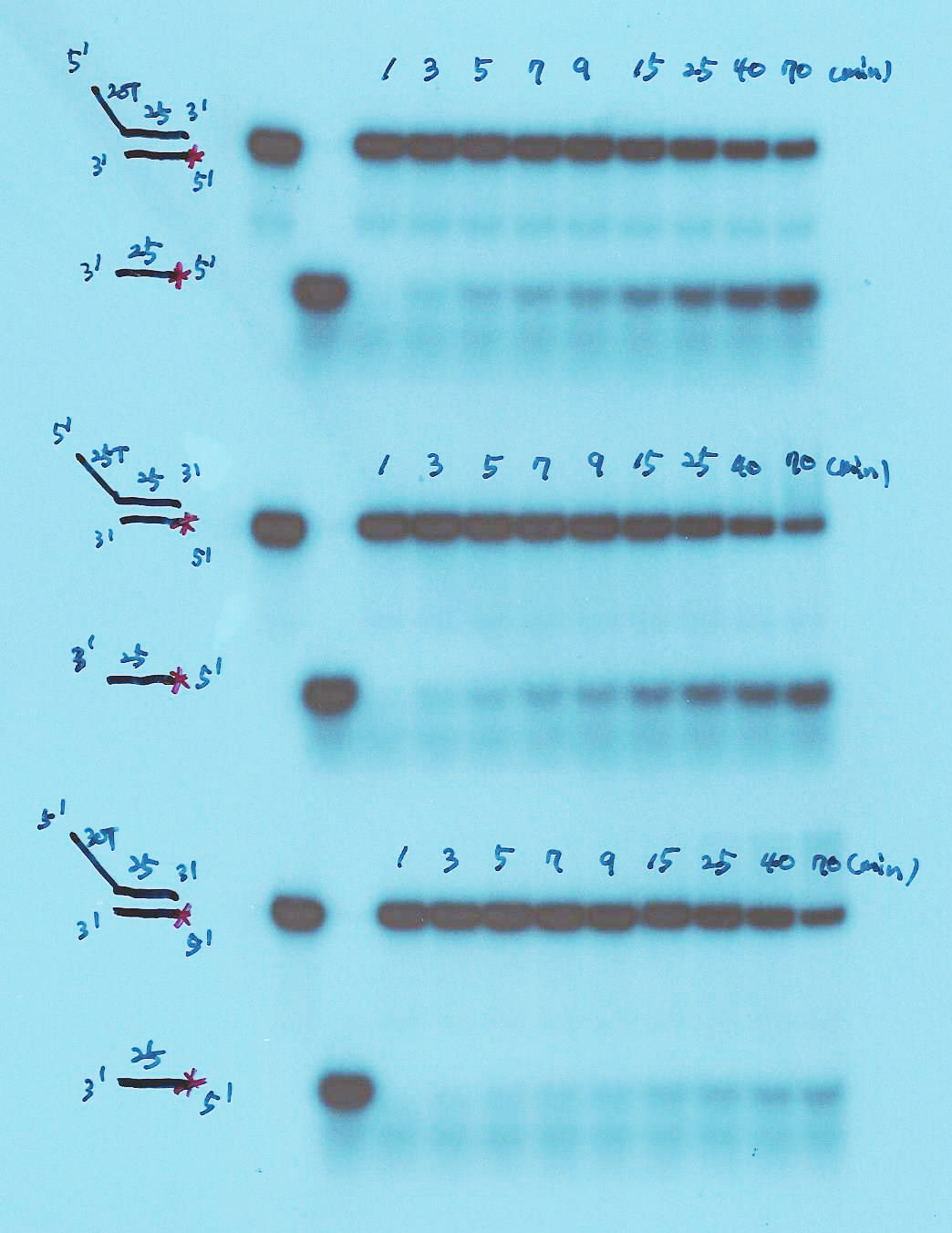


**Figure 4.** Substrate specificity of helicase nsP13 in the unwinding of duplex substrates.

Gel retardation assay of nsP13 and duplex RNA (30U/25D) and DNA (30T/25D) substrates


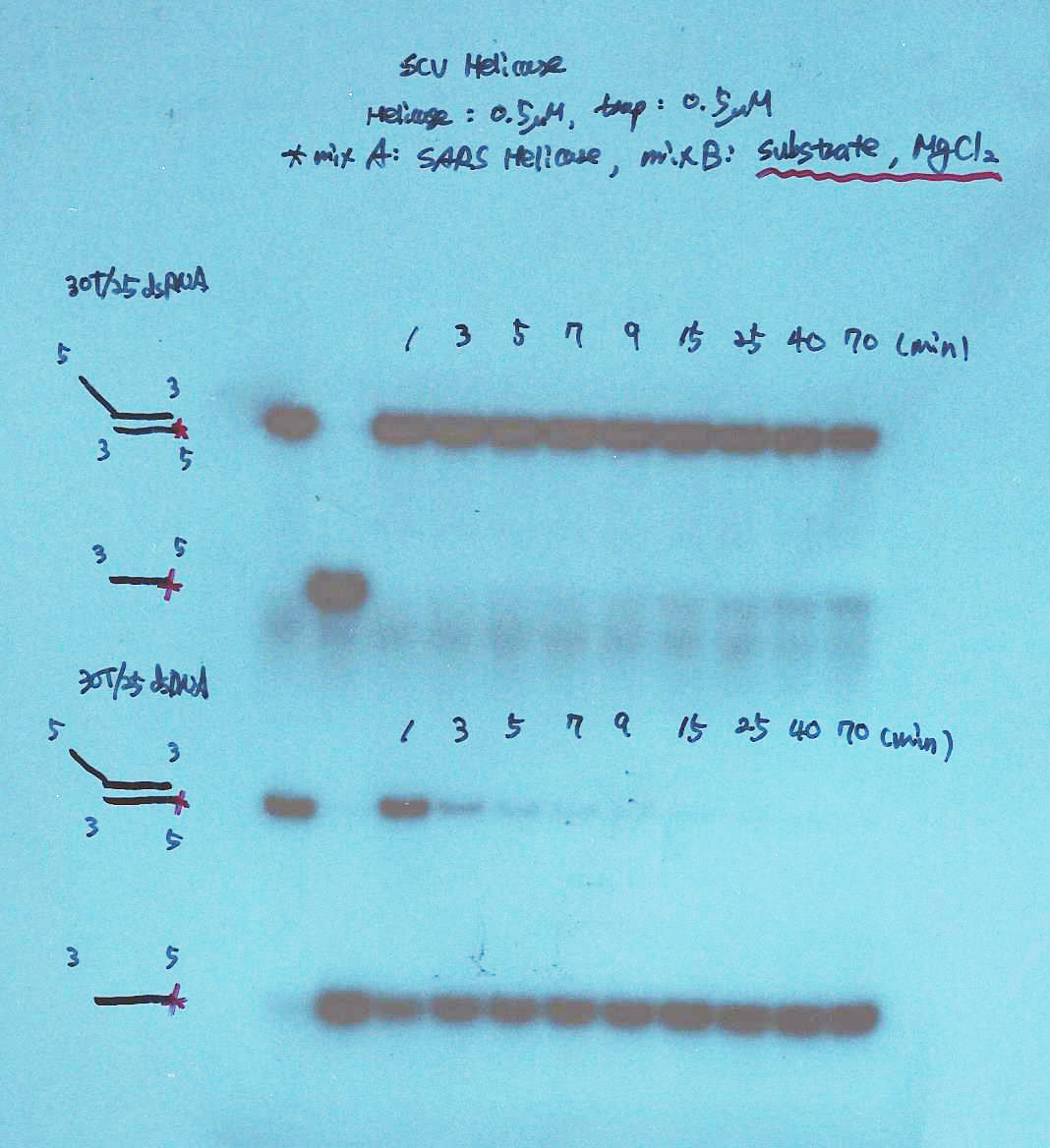


**Figure 5.** Difference in the substrate-dependent binding affinity of helicase nsP13.

1. Gel retardation assay of nsP13 and duplex RNA substrates: 30U/25D RNA

**
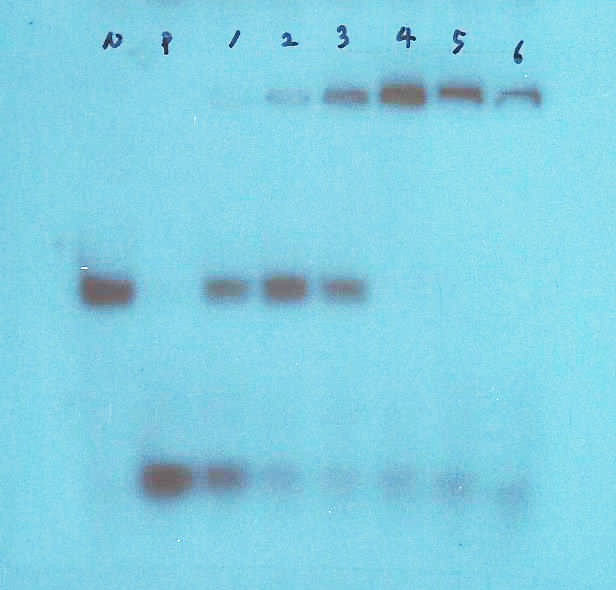
**

1. Gel retardation assay of nsP13 and duplex DNA substrates: 30T/25D DNA


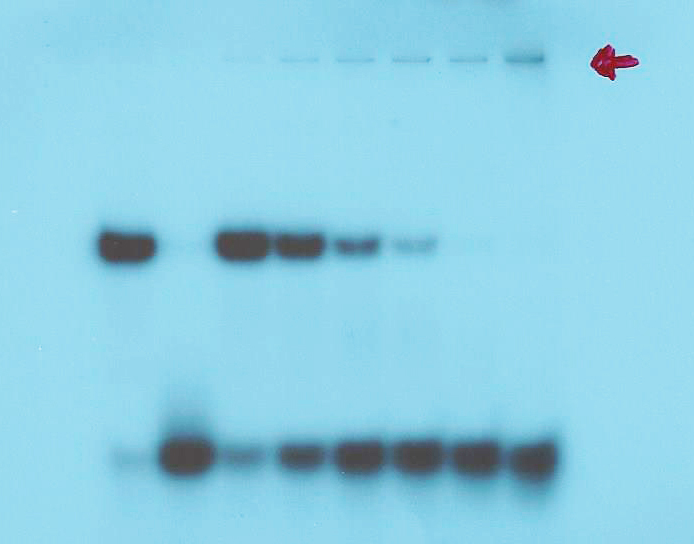


**Figure 6.** Additional ATP requirement to promote ATP-dependent translocation of helicase nsP13.

1. Gel retardation assay of nsP13 and duplex RNA substrates: 20U/25D RNA


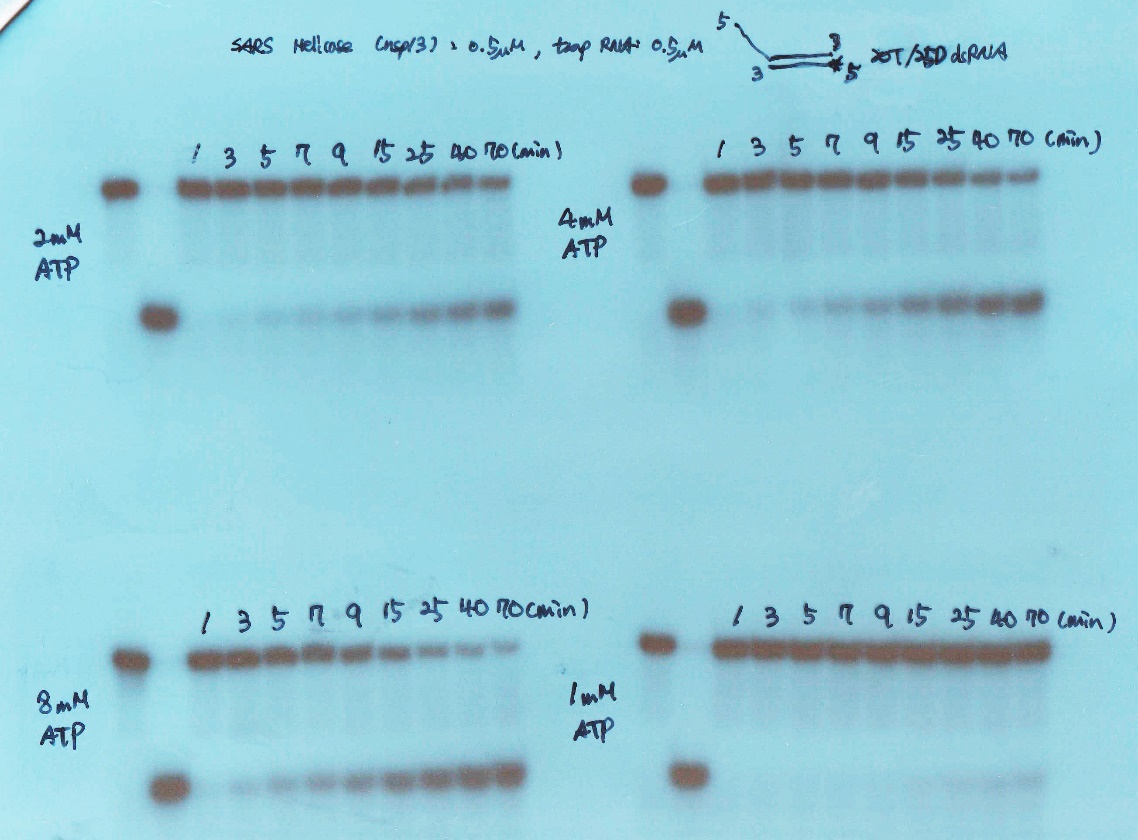


1. Gel retardation assay of nsP13 and duplex RNA substrates: 30U/25D RNA


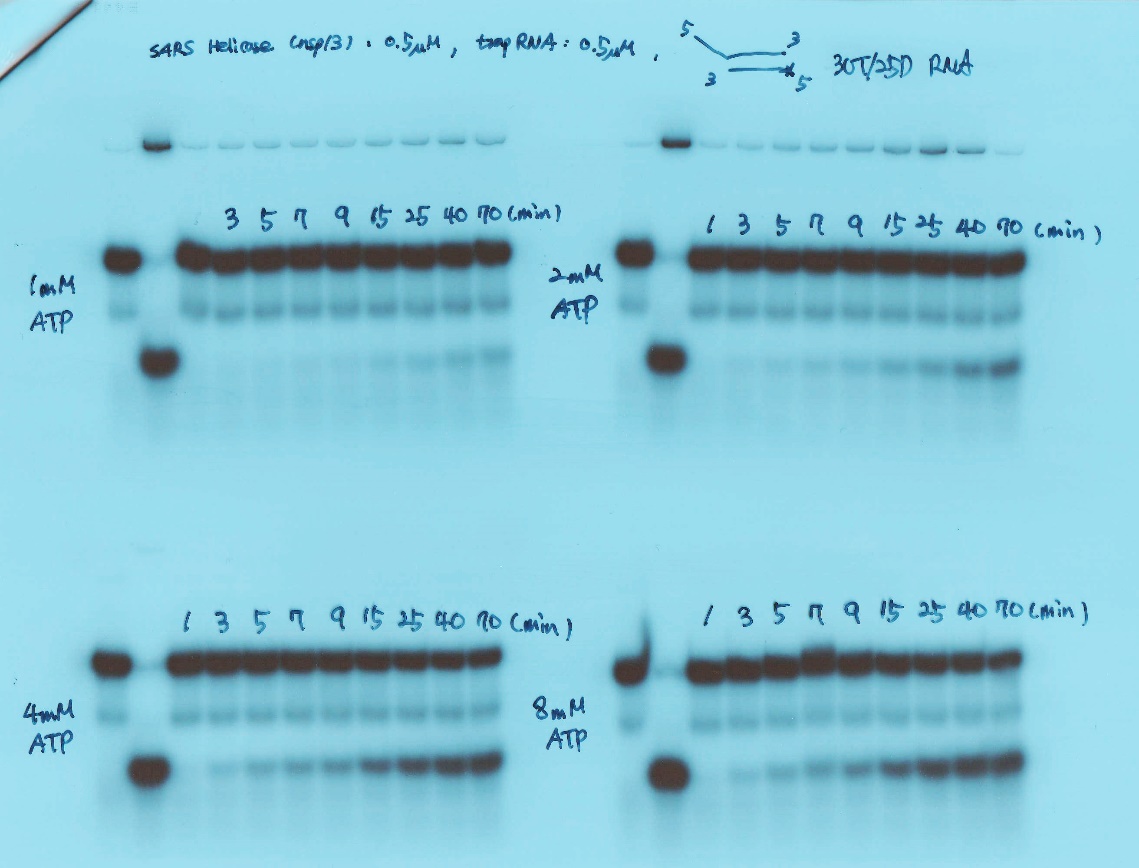


**Figure 7.** Enhanced duplex RNA unwinding by the cooperative translocation of helicase nsP13 under adequate ATP concentrations.

Gel retardation assay of nsP13 and duplex RNA substrate (30U/25D RNA)


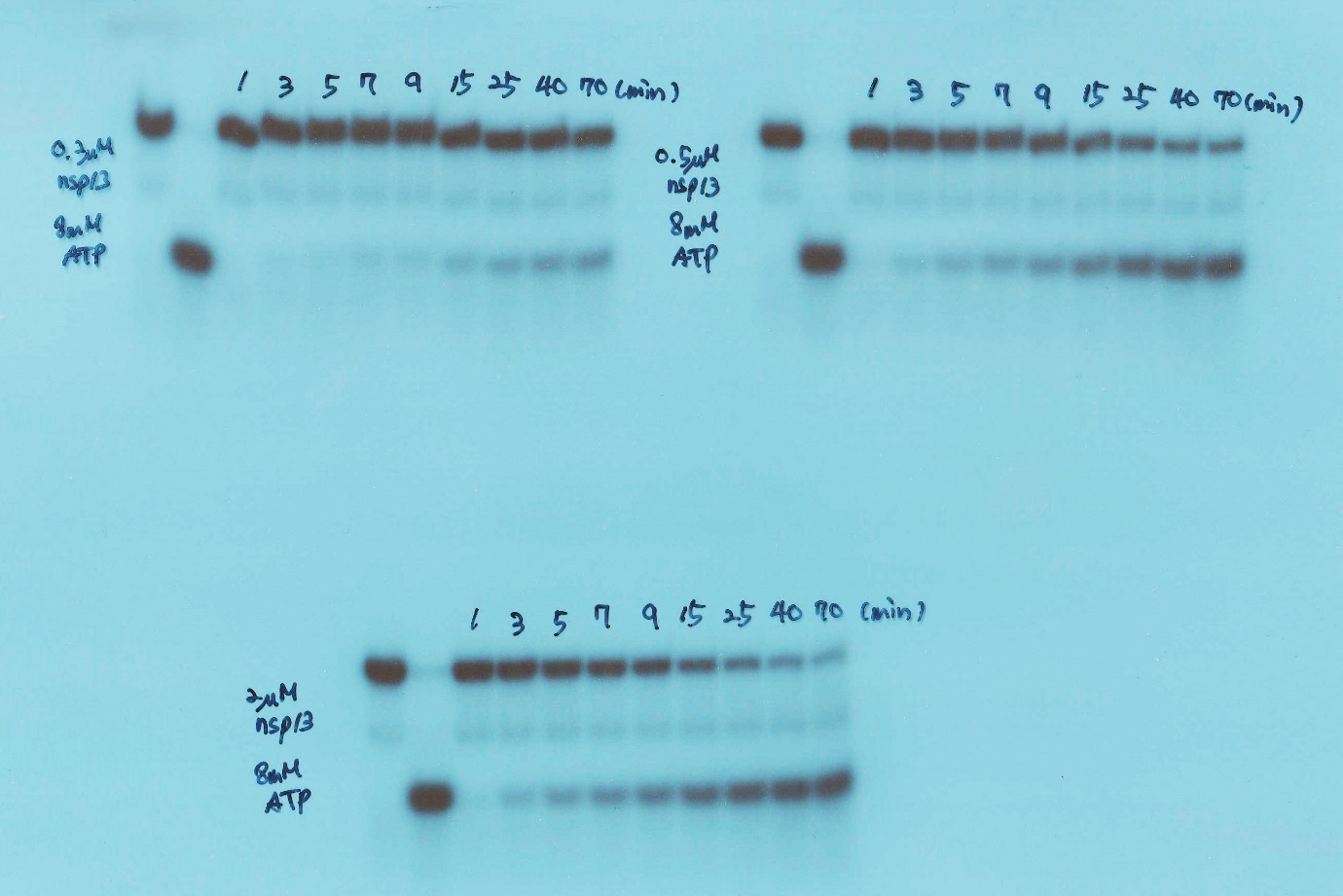


**Supplementary Figure 1**. Unwinding of duplex RNA (20U/15D RNA) with trap RNA of various concentrations.

1. Gel retardation assay of nsP13 and duplex RNA substrates.: 20U/15D RNA


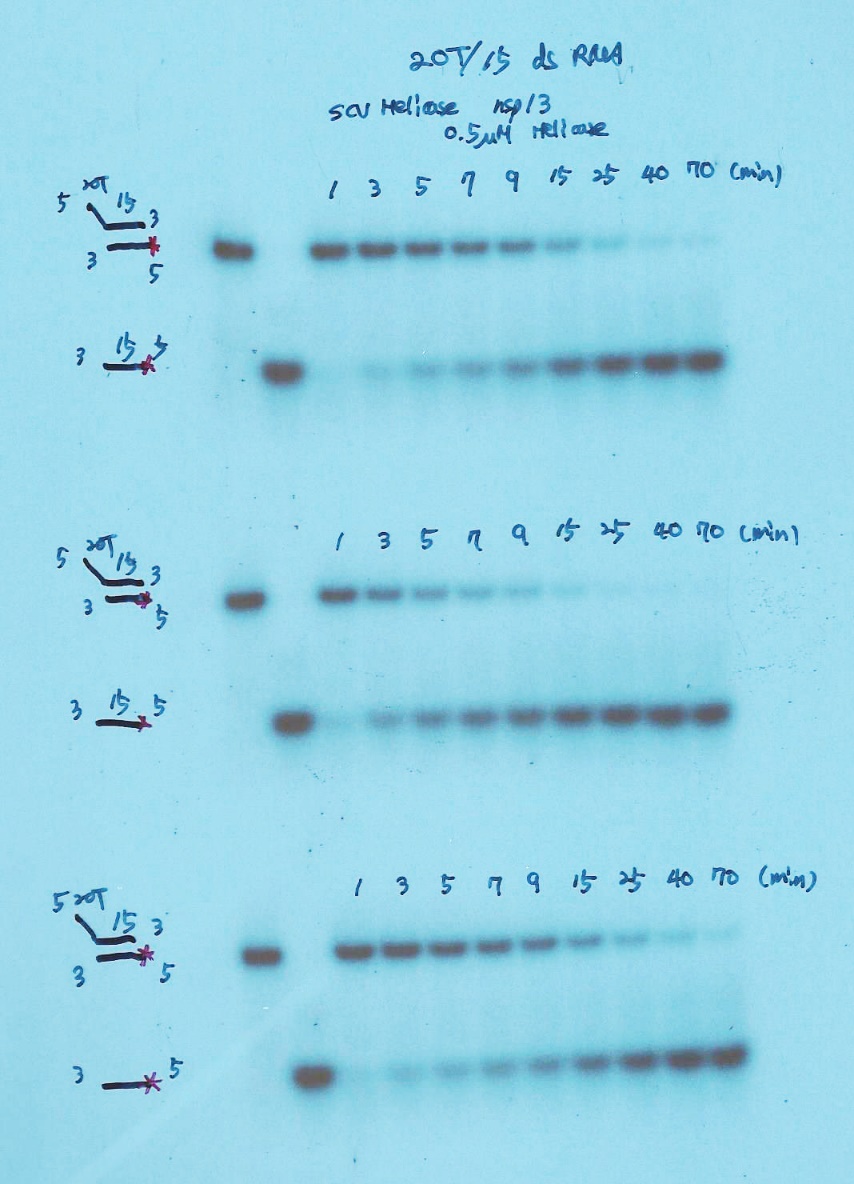


**Supplementary Figure 2.** Unwinding of duplex RNA substrates by varying helicase nsP13 concentrations.

1. Gel retardation assay of nsP13 and duplex RNA substrates.: 20U/15D RNA


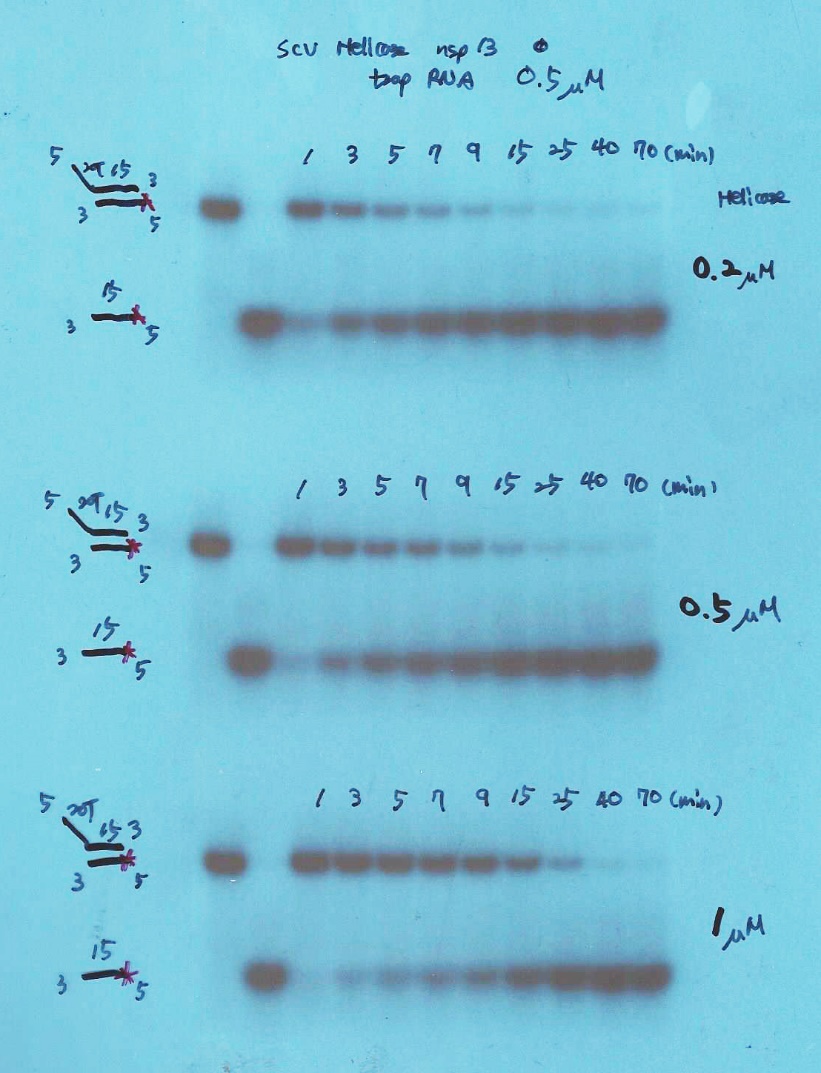


1. Gel retardation assay of nsP13 and duplex RNA substrates.: 20U/25D RNA


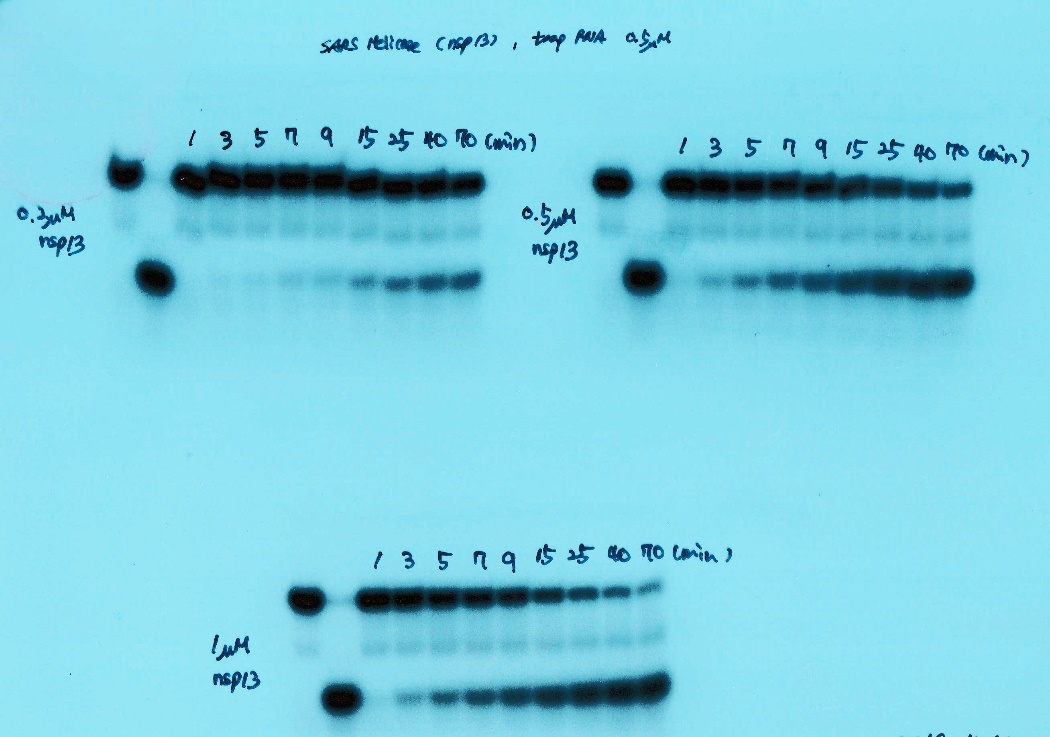


**Supplementary Figure 3.** Unwinding activity of duplex RNA with ATP and ATP analog (AMP-PNP).

20U/25D RNA: 2 mM ATP

*
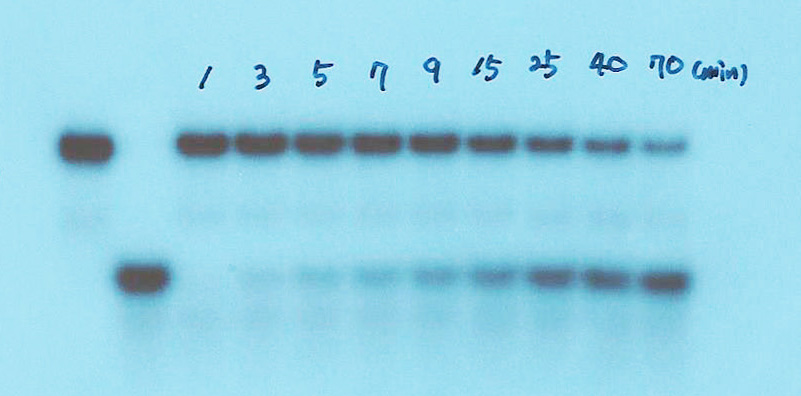
*

2OU/25D RNA: 2 mM AMP-PNP


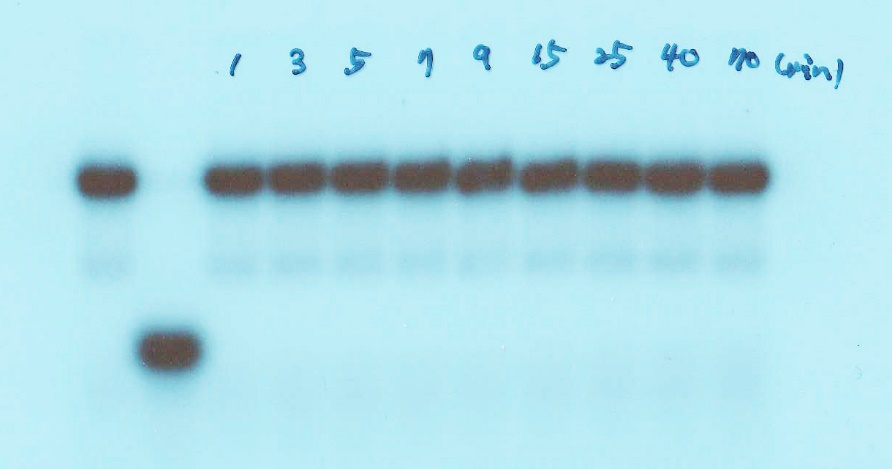


**Supplementary Figure 4.** Unwinding of duplex DNA substrates with different lengths of 5′-ss tail by helicase nsP13.

1. Gel retardation assay of nsP13 and duplex DNA substrates (20T, 25, and 30T/25D DNA).


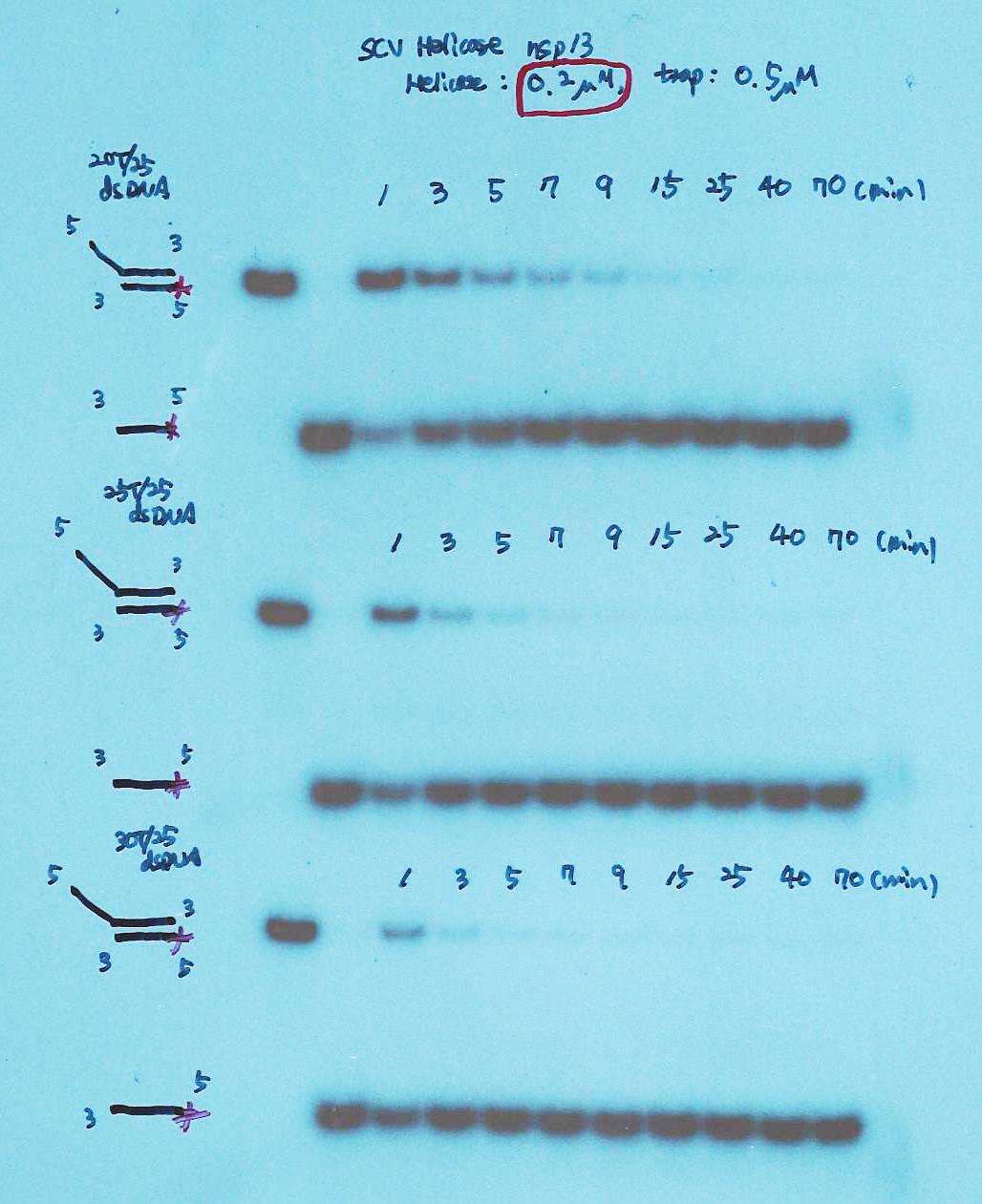


**Supplementary Figure 5.** Unwinding of duplex RNA substrates with 5′-ss tails of different lengths using low concentration of nsP13.


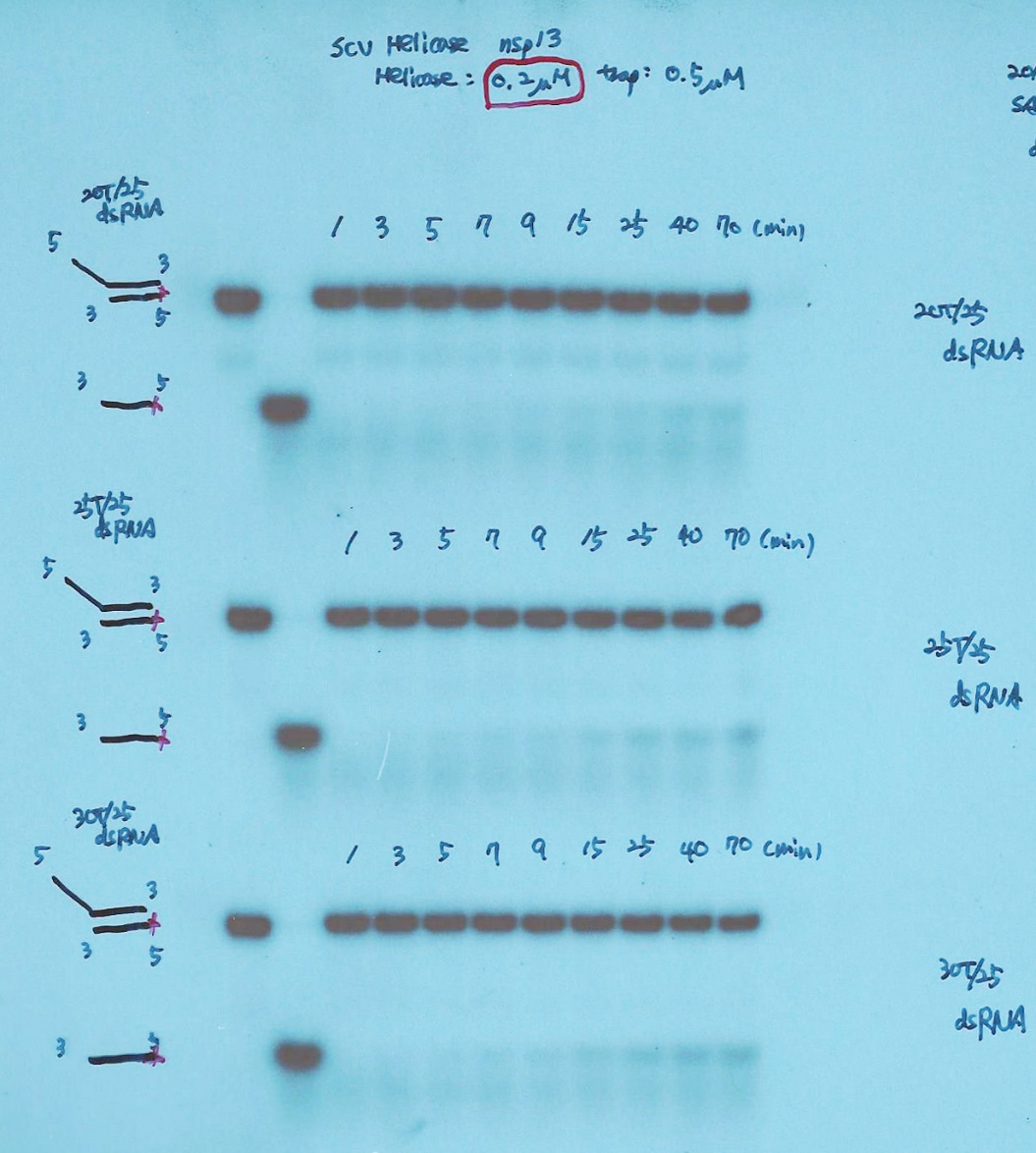


**Supplementary Figure 6.** Difference in the 5′-ss tail length-dependent binding affinity of helicase nsP13.


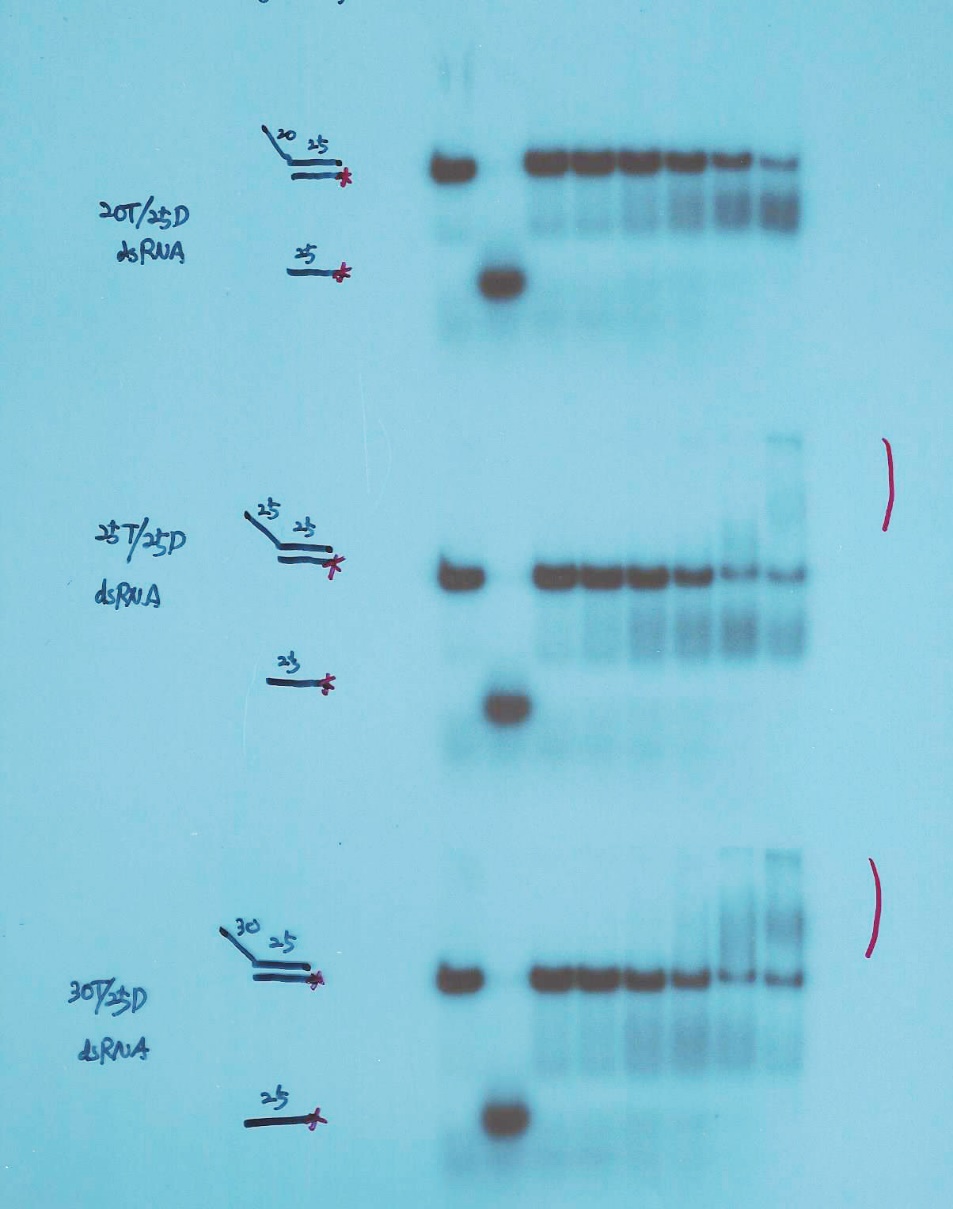


**Supplementary Figure 7.** Unwinding of duplex DNA substrates with 5′-ss tail of different lengths.

Gel retardation assay of nsP13 and duplex DNA substrates


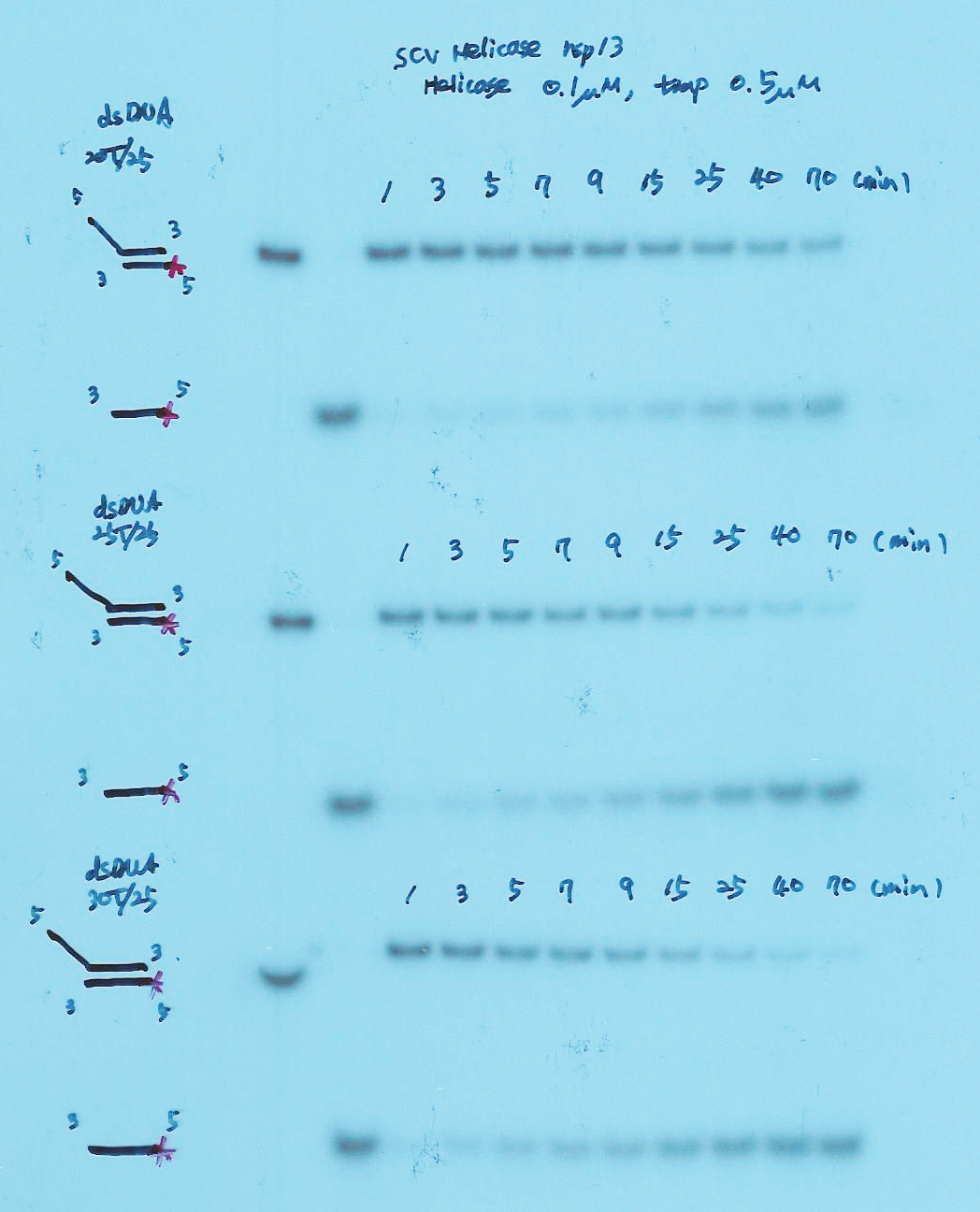


**Supplementary Figure 8.** Inhibition in duplex DNA unwinding of helicase nsP13 by increased ATP concentrations.

1. Gel retardation assay of nsP13 and duplex DNA substrates.: 20T/25D DNA


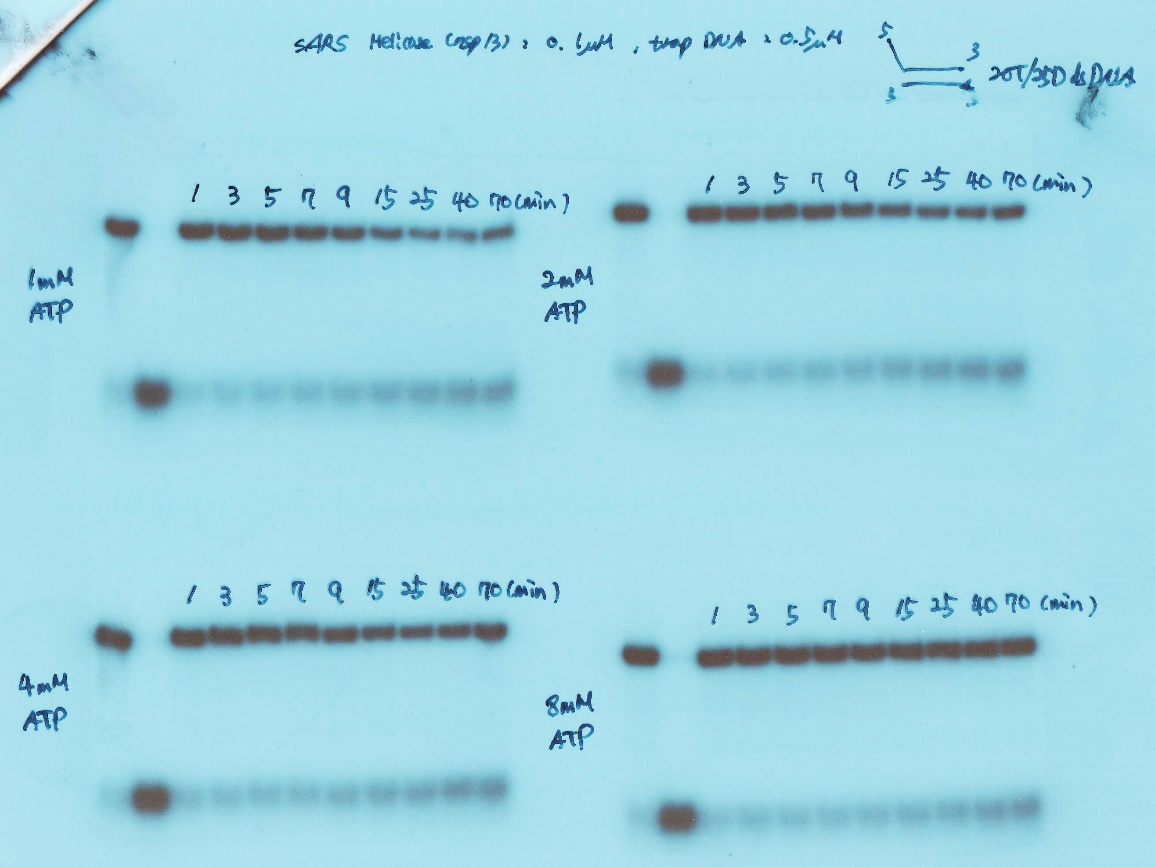


1. Gel retardation assay of nsP13 and duplex DNA substrates.: 30T/25D DNA


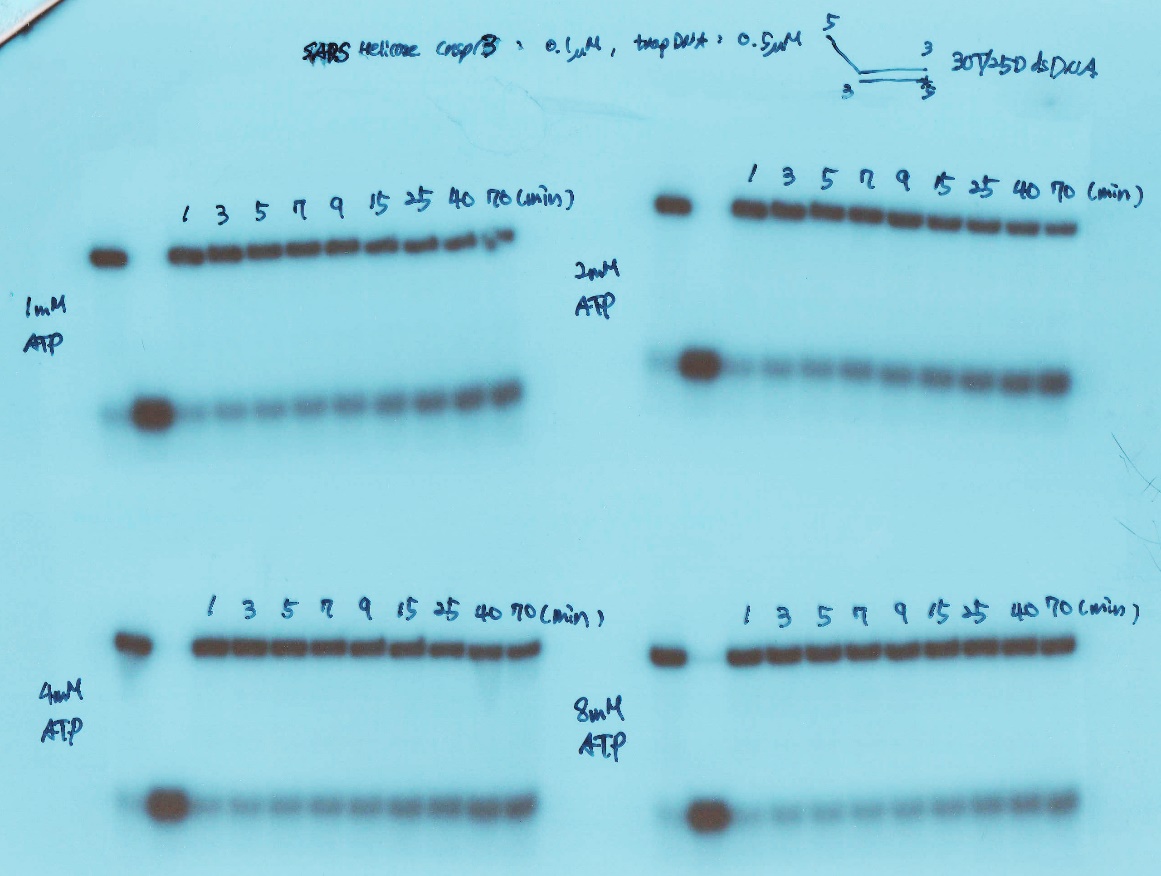

Supplement: Supplementary file 1 — Supplement information. [file 41598_2020_61432_MOESM1_ESM.docx]
